# Task force Guideline of Brazilian Society of Otology ‒ hearing loss in children – Part I ‒ Evaluation

**DOI:** 10.1016/j.bjorl.2022.11.002

**Published:** 2022-11-28

**Authors:** Vagner Antonio Rodrigues Silva, Henrique Furlan Pauna, Joel Lavinsky, Miguel Angelo Hyppolito, Melissa Ferreira Vianna, Mariana Leal, Eduardo Tanaka Massuda, Rogério Hamerschmidt, Fayez Bahmad Jr, Renato Valério Cal, André Luiz Lopes Sampaio, Felippe Felix, Carlos Takahiro Chone, Arthur Menino Castilho

**Affiliations:** a Universidade Estadual de Campinas (Unicamp), Faculdade de Ciências Médicas, Departamento de Otorrinolaringologia e Cirurgia de Cabeça e Pescoço, Campinas, SP, Brazil; b Hospital Universitário Cajuru, Departamento de Otorrinolaringologia, Curitiba, PR, Brazil; c Universidade Federal do Rio Grande do Sul (UFRGS), Departamento de Cirurgia, Porto Alegre, RS, Brazil; d Universidade de São Paulo (USP), Faculdade de Medicina de Ribeirão Preto, Departamento de Oftalmologia, Otorrinolaringologia e Cirurgia de Cabeça e Pescoço, Ribeirão Preto, SP, Brazil; e Irmandade Santa Casa de Misericórdia de São Paulo, Departamento de Otorrinolaringologia, São Paulo, SP, Brazil; f Universidade Federal de Pernambuco (UFPE), Departamento de Cirurgia, Recife, PE, Brazil; g Universidade Federal do Paraná (UFPR), Hospital de Clínicas, Departamento de Otorrinolaringologia e Cirurgia de Cabeça e Pescoço, Curitiba, PR, Brazil; h Universidade de Brasília (UnB), Programa de Pós-Graduação em Ciências da Saúde, Brasília, DF, Brazil; i Instituto Brasiliense de Otorrinolaringologia (IBO), Brasília, DF, Brazil; j Centro Universitário do Estado do Pará (CESUPA), Departamento de Otorrinolaringologia, Belém, PA, Brazil; k Universidade de Brasília (UnB), Faculdade de Medicina, Laboratório de Ensino e Pesquisa em Otorrinolaringologia, Brasília, DF, Brazil; l Universidade Federal do Rio de Janeiro (UFRJ), Hospital Universitário Clementino Fraga Filho (HUCFF), Departamento de Otorrinolaringologia, Rio de Janeiro, RJ, Brazil

**Keywords:** Hearing loss, Children, Guidelines, Screening, Diagnosis, Intervention

## Abstract

**Objectives:**

To provide an overview of the main evidence-based recommendations for the diagnosis of hearing loss in children and adolescents aged 0 to 18 years.

**Methods:**

Task force members were educated on knowledge synthesis methods, including electronic database search, review and selection of relevant citations, and critical appraisal of selected studies. Articles written in English or Portuguese on childhood hearing loss were eligible for inclusion. The American College of Physicians’ guideline grading system and the American Thyroid Association’s guideline criteria were used for critical appraisal of evidence and recommendations for therapeutic interventions.

**Results:**

The evaluation and diagnosis of hearing loss: universal newborn hearing screening, laboratory testing, congenital infections (especially cytomegalovirus), genetic testing and main syndromes, radiologic imaging studies, vestibular assessment of children with hearing loss, auditory neuropathy spectrum disorder, autism spectrum disorder, and noise-induced hearing loss.

**Conclusions:**

Every child with suspected hearing loss has the right to diagnosis and appropriate treatment if necessary. This task force considers 5 essential rights: (1) Otolaryngologist consultation; (2) Speech assessment and therapy; (3) Diagnostic tests; (4) Treatment; (5) Ophthalmologist consultation.

## Introduction

Approximately 360 million people (5% of the world’s population) have disabling hearing loss, and nearly 32 million of them are children.^1^ Childhood hearing impairment may cause language developmental delays, cognitive deficits, behavioral and emotional disorders, poor school performance, and lack of social interaction.^2^ A study showed that the prevalence of psychiatric disorders in a group of children with profound hearing loss reached 50%.^3^ The earlier children with hearing loss are identified and the earlier they receive adequate treatment, the greater the possibility for them to develop language and the lower the impact on their quality of life.^4^

The age at which hearing loss is detected has decreased since Universal Newborn Hearing Screening (UNHS) is mandatory in Brazil, in accordance with law number 12,303 of 2010.^5^ Historically, moderate-to-severe hearing loss in young children would go undetected until delayed language acquisition was observed. It was not uncommon for the diagnosis of mild hearing loss and unilateral hearing loss to be delayed until children reached school age.^6^

The World Health Organization (WHO) estimates that approximately 60% of the causes of hearing loss in children are preventable. More than 30% of cases are caused by infections such as rubella, Cytomegalovirus (CMV), mumps, meningitis, measles, and chronic ear infections. Meningitis and rubella together account for more than 19% of cases of childhood hearing loss. Most of these infections can be prevented by vaccination and improved socioeconomic and sanitation conditions. Acute middle ear infections and secretory otitis should be treated immediately with medical or surgical interventions. Perinatal and neonatal complications, such as lack of oxygen, low birth weight, prematurity, and jaundice, account for 17% of cases of childhood hearing loss. Such complications can be avoided by improving mother and child health practices.^1^ Noise exposure is another important factor. A study evaluating 5249 US adolescents aged 12 to 19 years showed that 15.9% had hearing deficits attributable to noise exposure.^7^ In the Netherlands, 14.2% of 5355 children aged 9 to 11 years were found to have hearing impairment relating to excessive headphone use.^8^

Since the 1970s in the United States, a multidisciplinary committee named Joint Committee on Infant Hearing (JCIH) has dedicated to detecting risk factors for children to develop hearing loss. There are periodic updates, and the latest statement, issued in 2019, highlighted the following^9^:

Perinatal or congenital:

• Neonatal Intensive Care Unit (ICU) for more than 5 days; Hyperbilirubinemia with exchange transfusion regardless of length of ICU stay; Asphyxia or hypoxic-ischemic encephalopathy; Extracorporeal Membrane Oxygenation (ECMO); Microcephaly; Syndromes associated with progressive hearing loss; Congenital or acquired hydrocephalus; Temporal bone abnormalities; In utero infections with syphilis, toxoplasmosis, rubella, CMV, herpes (STORCH), or Zika; Family history of early, progressive, or delayed permanent childhood deafness.
• Delayed: Infections that lead to deafness, such as bacterial or viral meningitis and encephalitis (especially herpes and chickenpox viruses); Chemotherapy; Family suspicion of deafness, speech or language impairment, and developmental delay or regression; Skull or temporal bone trauma.

The JCIH states that children at risk of hearing loss need to be closely monitored, even after undergoing UNHS. This ensures the identification of hearing loss as early as possible and avoids treatment delays.^10^ Studies^11^, ^12^ suggest that the prevalence of permanent hearing loss increases during childhood and that hearing loss occurs sometime after childhood in up to 25%‒50% of children with risk factors.

Almost half of hard-of-hearing children will experience hearing deterioration during childhood.^13^ It is also important to monitor children with early hearing loss who are at risk for hearing deterioration (progressive hearing loss) so that appropriate intervention can be initiated in a timely manner. Thus, surveillance protocols are recommended as part of a comprehensive UNHS program but have not yet been universally applied.^14^

## Etiologies

For didactic purposes, the main causes of hearing loss in children can be divided into congenital and acquired. The congenital causes include diseases that manifest within the neonatal period (up to 28 days of life) or later (after 28 days of life).

### Neonatal causes

The estimated prevalence of congenital profound bilateral hearing loss is 1 in 1000 newborns.^2^ Another 1 to 2 in 1000 newborns have mild-to-moderate bilateral hearing loss or unilateral hearing loss of any degree of severity.^15^ The most common causes of congenital sensory hearing loss are infections (syphilis, toxoplasmosis, rubella, and CMV), inner ear malformations (30% to 40%), and genetic causes (50%).^16^ Rubella was once the most common viral cause of congenital sensory hearing loss, but vaccination has made it rare today.^17^ Congenital syphilis, which had declined for decades and remains uncommon, is on the rise, with an incidence of 23.3 per 100,000 live births in 2017.^18^ Nearly 50% of children with congenital hearing loss do not have a specific diagnosis.

The Zika virus is transmitted by the *Aedes aegypti* mosquito and can cross the placental barrier and infect the developing fetus. Congenital Zika Syndrome (CZS) may cause microcephaly, reduced brain parenchyma, and visual and hearing impairment. Approximately 7% of infants with CZS have sensorineural hearing loss.^19^ Although the main concern with Zika virus infections is CZS, children infected with Zika virus after the neonatal period are at risk for Guillain-Barré syndrome and transient sensorineural hearing loss.

The incidence of congenital hearing loss is higher in premature children and becomes lower with increasing gestational age and birth weight (1.2% to 7.5% born at 24‒31 weeks and 1.4% to 4.8% with birth weight 750 to 1500 g). Hearing loss affects 1.2% to 7.5% of infants in neonatal ICUs.^20^ Interventions such as assisted ventilation, prolonged hospital stays (≥12 days), central venous access, and antibiotic use in the neonatal ICU increase the likelihood of hearing loss.

GJB2, the first gene to be implicated in nonsyndromic human deafness, was discovered in 1997. It is the gene most related to hearing loss. Since then, more than 130 genes and nearly 8000 gene variations have been identified and are associated with severe-to-profound nonsyndromic sensorineural hearing loss. These variations differ significantly based on the ethnic population examined. There are also over 600 clinical syndromes that include deafness. In many of them, deafness is the first clinical feature to manifest.^21^ According to estimates, there are over 1000 genes that can cause hearing impairment.^22^

Some syndromes such as coloboma, heart disease, choanal atresia, retarded growth and development, genital hypoplasia, ear anomalies/deafness (CHARGE), associated with genetic causes, may also cause hearing loss due to middle or inner ear malformations.^23^

### Delayed onset causes

Some children may develop deafness after the neonatal period, and this will not be detected by current UNHS. Importantly, a significant number of children who develop prelingual deafness after the neonatal period go undetected by currently implemented UNHS. Dedhia et al.^24^ estimated that nearly 25% of all children with sensorineural hearing loss are not identified by UNHS and that two-thirds of them had severe-to-profound deafness. Some syndromes can only be identified over time.

Pendred syndrome is associated with recessive variants in the SLC26A4 gene. It is the most common syndromic form of hereditary sensory hearing loss and is associated with thyroid dysfunction, goiter, Enlarged Vestibular Aqueduct (EVA), and cochlear incomplete partition type II (Mondini deformity).^25^

Usher syndrome is also autosomal recessive and has 3 clinical types, associated with at least 9 genes that are differentiated by the severity of hearing loss, vestibular dysfunction, and age at onset of vision loss.^26^ Alport syndrome is an X-linked (80%) or recessive (depending on the gene) disorder resulting in kidney failure, eye abnormalities (lenticonus, subcapsular cataract, retinopathy), and progressive sensory hearing loss usually detected in late childhood.^27^

Delayed-onset hearing loss may also occur after congenital infections. Historically, prenatal exposure to STORCH agents was a common cause of congenital hearing loss. However, the epidemiology of these agents has changed, and congenital CMV (cCMV) currently is a major cause of delayed-onset hearing loss in many countries. The prevalence of cCMV infection is 0.4% to 2.3% of all newborns. Of infants with confirmed congenital loss, 6% to 7% have cCMV.^28^ However, up to 43% of infants with cCMV initially undergo UNHS but will later experience sensory hearing loss.^28^

### Acquired causes

Hearing loss in children may have multiple etiologies over time, such as temporal bone fractures, infections, exposure to ototoxic drugs, autoimmune diseases, and noise exposure. The estimated prevalence of hearing loss in children up to 18 years of age is 18%.^29^

Trauma may cause conductive, mixed, or sensorineural hearing loss, depending on the location and type of temporal bone injury. Up to 82% of children with temporal bone fractures will have hearing loss at presentation; of these cases, 56% will be conductive, 17% will be sensorineural, and 10% will be mixed.^30^ Conductive hearing loss may result from tympanic membrane perforation or ossicular chain injury. Temporal bone fractures may damage the cochlea and the cochlear nerve, or cause a labyrinthine fistula, which often leads to severe-to-profound sensorineural hearing loss.^31^ Temporal bone concussions without fracture may also result in temporary or permanent sensory hearing loss.^32^

Infectious causes of sensory hearing loss include measles, mumps, varicella-zoster virus, Lyme disease, bacterial meningitis, and, rarely, otitis media. Measles and mumps with subsequent hearing loss are more common in unvaccinated children.^33^ Meningitis in children is the most common postnatal cause of acquired bilateral hearing loss. Meningogenic labyrinthitis (with or without new bone formation) is most often due to bacterial meningitis and is usually bilateral. The offending pathogens are believed to invade the membranous labyrinth through the cochlear aqueducts or the lamina cribrosa of the vestibule, resulting in suppurative labyrinthitis. Three radiologic stages are described in labyrinthitis: acute stage, fibrous stage, and labyrinthitis ossificans, shown by both Computed Tomography (CT) and Magnetic Resonance Imaging (MRI).^30^

Hearing loss resulting from bacterial meningitis may be progressive and is more common after *Streptococcus pneumoniae* infections that can cause ossification of the labyrinth. Cochlear implantation is indicated as soon as possible.^34^ Acute and chronic otitis media may also cause hearing loss. They are usually conductive and can be resolved with antibiotic therapy or surgery.

The following drugs are known to be ototoxic and cause permanent hearing loss: aminoglycosides, chemotherapy drugs (especially cisplatin), and loop diuretics. Radiotherapy involving the temporal bones, associated with cisplatin, increases the risk of sensorineural hearing loss, which may manifest a few years after the end of treatment.^35^ Although aminoglycoside-induced ototoxicity is strongly related to serum drug level and cumulative dosage, genetic susceptibility to ototoxicity is well described (including mitochondrial gene mutations, especially RNA 1555A>G and 1494C>T in mitochondrial 12S ribosomal RNA), with hearing loss occurring after even a single dose in some patients who have these mutations.^36^ Other drugs such as salicylates and macrolides may cause hearing loss that is usually reversible.^37^ Close monitoring of serum drug doses and levels can decrease the chance of inner ear injury.

Autoimmune hearing loss is due to either primary autoimmune dysfunctions in the inner ear or systemic autoimmune diseases such as Cogan syndrome (interstitial keratitis, progressive hearing loss, and vestibular dysfunction).^38^ Hearing loss is often rapidly progressive and sometimes responds to immunosuppressants.

According to the WHO, 1.1 billion young people are at risk of hearing loss due to prolonged and excessive exposure to loud sounds.^39^ Children and adolescents are at higher risk of developing hearing loss because of frequent exposure to loud music during leisure activities, on transport services, and while playing sports.^40^, ^41^ Headphones improve the listening experience but also increase the risk of noise-induced hearing loss. The use of portable music players such as MP3 players, iPods, smartphones, and similar devices has increased worldwide over the past few decades.^42^

## Hearing tests

The tests used for identifying neonatal hearing loss are transient-evoked and distortion-product Otoacoustic Emissions (OAE) and Auditory Brainstem Response (ABR) (click, tone-burst, chirp, steady-state). These electrophysiologic tests are suitable for children because they are considered objective and performed under sedation, but their interpretation is examiner dependent. These tests will be described later, when UNHS is discussed.

In children aged 7 years or over, subjective tests can be used, such as audiometry. This test is dependent on the child’s response and the examiner’s experience.

Hearing loss is defined as compromised ability to hear sounds at thresholds considered normal. For children, an average pure-tone threshold higher than 15 dB HL at 500, 1000, 2000 and 4000 Hz is considered outside the normal reference range. The severity of hearing loss is categorized in Table 1.

**Table 1:** Severity of hearing loss in children up to 7 years of age, considering tone thresholds between 500 and 4000 Hz.

Severity of hearing loss	Hearing thresholds (dB)	
	Northern and Downs, 2002^43^	WHO, 2016^1^
Normal	0 to 15	0 to 15
Slight	16 to 25	NA
Mild	26 to 30	16 to 30
Moderate	31 to 50	31 to 60
Severe	51 to 70	61 to 80
Profound	>71	>81

dB, Decibel; WHO, World Health Organization; NA, Not Applicable. The term “slight” is not used in WHO classification.

## Objective

This systematic review has the purpose to provide an overview of the evidence-based recommendations for the diagnosis of hearing loss in children and adolescents aged 0 to 18 years.

## Methods

Between April 28 and 29, 2022, a task force consisting of otolaryngologists, otology specialists, Brazilian Society of Otology (Sociedade Brasileira de Otologia, SBO) directors, and some SBO members met (in person and remotely) to discuss the topic of this guideline. Each participant in this meeting was tasked with giving a 15-min evidence-based lecture on one of the suggested topics. After the lecture, the participants discussed the topic until reaching a consensus. Each author was asked to write a text with the current literature on the topic, based on evidence and containing the elements discussed during the meeting. A rapporteur prepared the final text, which was reviewed by 4 additional coauthors and the Brazilian Journal of Otorhinolaryngology (BJORL) editor.

This guideline is not intended to be a substitute for individual professional judgment. Physicians should always act and decide in a way that they believe is best for their patients, regardless of guideline recommendations. They should also operate within their scope of practice and in accordance with their training. The guidelines represent the best judgment of a team of experienced physicians addressing the scientific evidence for a given topic.

The grading system of the American College of Physicians (ACP) was used in this guideline, relating to critical appraisal and recommendations on therapeutic interventions^44^ (Table 2, Table 3). An important component of this guideline was judged to be critical appraisal of diagnostic testing studies. However, the ACP guideline grading system was not designed for this purpose.^45^, ^46^, ^47^

**Table 2:** Interpretation of the American College of Physicians’ Guideline Grading System (for Therapeutic Interventions).

Recommendation	Clarity of risk/benefit	Implications
Strong recommendation	Benefits clearly outweigh harms and burdens, or vice versa.	Patients: Most would want course of action; a person should request discussion if an intervention is not offered.
		Clinicians: Most patients should receive the recommended course of action.
		Policymakers: The recommendation can be adopted as policy in most circumstances.
Weak recommendation	Benefits closely balanced with harms and burdens.	Patients: Many would want course of action, but some may not; the decision may depend on individual circumstances.
		Clinicians: Different choices will be appropriate for different patients; the management decision should be consistent with patients’ preferences and circumstances.
		Policymakers: Policymaking will require careful consideration and stakeholder input.
No recommendation	Balance of benefits and risks cannot be determined.	Decisions based on evidence cannot be made.

**Table 3:** Recommendations (for therapeutic interventions) based on strength of evidence.

Recommendation and evidence of quality	Description of supporting evidence^a^	Interpretation
Strong recommendation		
High-quality evidence	RCT without important limitations or overwhelming evidence from observational studies.	Can apply to most patients in most circumstances without reservation.
Moderate-quality evidence	RCT with important limitations or strong evidence from observational studies.	Can apply to most patients in most circumstances without reservation.
Low-quality evidence	Observational studies/case studies.	May change when higher-quality evidence becomes available.
Weak recommendation		
High-quality evidence	RCT without important limitations or overwhelming evidence from observational studies.	Best action may differ based on circumstances or patients’ values.
Moderate-quality evidence	RCT with important limitations or strong evidence from observational studies.	Best action may differ based on circumstances or patients’ values.
Low-quality evidence	Observational studies/case studies.	Other alternatives may be equally reasonable.
Insufficient	Evidence is conflicting, of poor quality, or lacking.	Insufficient evidence to recommend for or against.

a This description of supporting evidence refers to therapy, therapeutic strategy, or prevention studies. The description of supporting evidence is different for diagnostic accuracy studies. RCT, Randomized Controlled Trial.

The American Thyroid Association (ATA) created a diagnostic test appraisal system that included consideration of the following methodological elements: consecutive recruitment of patients representative of clinical practice, use of an appropriate reference gold standard, directness of evidence (target population of interest, testing procedures representative of clinical practice, and relevant outcomes), precision of diagnostic accuracy measures (confidence intervals for estimates such as sensitivity and specificity), and consistency of results across studies using the same test that was also used in this guideline^46^ (Table 4, Table 5).

**Table 4:** Interpretation of the American Thyroid Association Guideline for Diagnostic Tests.

Recommendation	Accuracy of diagnostic information versus risks and burden of testing	Implications
Strong recommendation	Knowledge of the diagnostic test result clearly outweighs risks and burden of testing or vice versa.	Patients: In the case of an accurate test for which benefits outweigh risks/burden, most would want the diagnostic test to be offered (with appropriate counseling). A patient should request discussion of the test if it is not offered. In contrast, for a test in which risks/burden outweigh the benefits, most patients should not expect the test to be offered.
		Clinicians: In the case of an accurate test for which benefits outweigh risks/burden, most patients should be offered the diagnostic test (and provided relevant counseling). Counseling about the test should include a discussion of the risks, benefits, and uncertainties related to testing (as applicable), as well as the implications of the test result. In contrast, for a test in which risks/burden outweigh the perceived benefits, most patients should not be offered the test, or if the test is discussed, the rationale against the test should, for the particular clinical situation, be explained.
		Policymakers: In the case of an accurate test for which benefits outweigh risks/burden, availability of the diagnostic test should be adopted in health policy. In contrast, for a test in which risks/burden outweigh the perceived benefits, some restrictions on circumstances for test use may need to be considered.
Weak recommendation	Knowledge of the diagnostic test result is closely balanced with risks and burden of testing.	Patients: Most would want to be informed about the diagnostic test, but some would not want to seriously consider undergoing the test; a decision may depend on the individual circumstances (e.g., risk of disease, comorbidities, or other), the practice environment, feasibility of optimal execution of the test, and consideration of other available options.
		Clinicians: Different choices will be appropriate for different patients, and counseling about the test (if being considered) should include a discussion of the risks, benefits, and uncertainties related to testing (as applicable), as well as the implications of the test result. The decision to perform the test should include consideration of the patients’ values, preferences, feasibility, and the specific circumstances. Counseling the patient on why the test may be helpful or not, in her/his specific circumstance, may be highly valuable in the decision-making process.
		Policymakers: Policymaking decisions on the availability of the test will require discussion and stakeholder involvement.
No recommendation	Balance of knowledge of the diagnostic test result cannot be determined.	Decisions on the use of the test based on evidence from scientific studies cannot be made.

**Table 5:** Recommendations (for diagnostic interventions) based on strength of evidence.

Recommendation and evidence of quality	Methodologic quality of supporting evidence	Interpretation
Strong recommendation		
High-quality evidence	Evidence from one or more well-designed nonrandomized diagnostic accuracy studies (i.e., observational ‒ cross-sectional or cohort) or systematic reviews/meta-analyses of such observational studies (with no concern about internal validity or external generalizability of the results).	Implies the test can be offered to most patients in most applicable circumstances.
Moderate-quality evidence	Evidence from nonrandomized diagnostic accuracy studies (cross-sectional or cohort), with one or more possible limitations causing minor concern about internal validity or external generalizability of the results.	Implies the test can be offered to most patients in most applicable circumstances without reservation.
Low-quality evidence	Evidence from nonrandomized diagnostic accuracy studies with one or more important limitations causing serious concern about internal validity or external generalizability of the results.	Implies the test can be offered to most patients in most applicable circumstances, but the utilization of the test may change when higher-quality evidence becomes available.
Weak recommendation		
High-quality evidence	Evidence from one or more well-designed nonrandomized diagnostic accuracy studies (i.e., observational ‒ cross-sectional or cohort) or systematic reviews/meta-analyses of such observational studies (with no concern about internal validity or external generalizability of the results).	The degree to which the diagnostic test is seriously considered may differ depending on circumstances or patients’ or societal values.
Moderate-quality evidence	Evidence from nonrandomized diagnostic accuracy studies (cross-sectional or cohort), with one or more possible limitations causing minor concern about internal validity or external generalizability of the results.	The degree to which the diagnostic test is seriously considered may differ depending on individual patients’/practice circumstances or patients’ or societal values.
Low-quality evidence	Evidence from nonrandomized diagnostic accuracy studies with one or more important limitations causing serious concern about internal validity or external generalizability of the results.	Alternative options may be equally reasonable.
Insufficient	Evidence may be of such poor quality, conflicting, lacking (i.e., studies not done), or not externally generalizable to the target clinical population such that the estimate of the true effect of the test is uncertain and does not permit a reasonable conclusion to be made.	Insufficient evidence exists to recommend for or against routinely offering the diagnostic test.

## Evidence-based evaluation and diagnosis of hearing loss in children

### Universal newborn hearing screening protocol

In the 2019 JCIH statement recommendations,^9^ there is a major concern with starting rehabilitation as early as possible. Screening is suggested to be done up to 1 month of age, diagnosis of hearing loss to be made up to 2 months of age, and rehabilitation to be started up to 3 months of age. This new goal has been established mainly for programs that already meet the 2007 recommendations^6^ of screening up to 1 month, diagnosis up to 3 months, and start of rehabilitation up to 6 months. This concern is based on the knowledge that early intervention is critical for successful rehabilitation outcomes. Delays have a negative impact not only on language development and communication but also on children’s well-being and cognition.

In Brazil, the reality is still far from ideal, despite a 2010 law^5^ defining the mandatory nature of UNHS. In a study on UNHS coverage in public maternity hospitals in the country, a 34.4% coverage was identified by 2018. There were large regional differences, with almost 70% coverage in the South region and only 21.9% in the North region in the same period.^48^

#### Newborns without risk factors for hearing loss

UNHS is essential for congenital hearing loss to be detected and rehabilitated as early as possible. Fifty percent of children with hearing loss at birth do not have any risk factors for hearing loss.^49^ Because UNHS should be done in all newborns before leaving the maternity hospital, it requires, in addition to appropriate equipment, qualified professionals available daily so that no diagnoses of hearing loss are missed.

Screening can be done using 2 methods, OAE or Automated ABR (AABR). Given the very low incidence of auditory neuropathy in children who are not in the ICU, both methods have been shown to be appropriate and are equivalent screening methods for hearing loss identification.^50^

A 2-stage UNHS procedure should be conducted. Children who “failed” the first evaluation should undergo a retest, which can be the same as before or another method. It is important that this retest is performed before 1 month of life, preferably before discharge from the maternity hospital if possible, and that there is no diagnostic delay in case of new “failed” retests. If the first and second evaluations fail, even in one of the ears, the child should be immediately referred for otolaryngologic evaluation and diagnostic testing.

There are, however, important differences between the 2 methods. The OAE test measures the response of the cochlea’s outer hair cells, while the ABR test reflects both cochlear status and auditory neural function that extends beyond the cochlea to the brainstem. However, both have shown to be adequate and equivalent for UNHS, despite having some limitations, such as the influence of responses due to middle ear problems.

UNHS may not identify mild losses (less than 25‒40 dB) more often when performed with AABR,^51^ while detection of auditory neuropathy may fail especially if screening is performed with OAE.^52^ In addition, late or progressive losses may not be detected, which makes follow-up with hearing and language developmental milestones essential. Regardless of UNHS outcomes, all infants and children should be routinely monitored for hearing, cognitive development, communication, achievement of educational milestones, general health, and well-being.^49^

#### Newborns at high risk for hearing loss

In newborns at high risk for hearing loss, as previously described, sensorineural deafness accounts for 2.5% to 10% and conductive hearing loss for 25% to 35%.^14^, ^53^, ^54^ The main causes are congenital or perinatal. Thus, UNHS is extremely important in this population.

**Figure 1:**
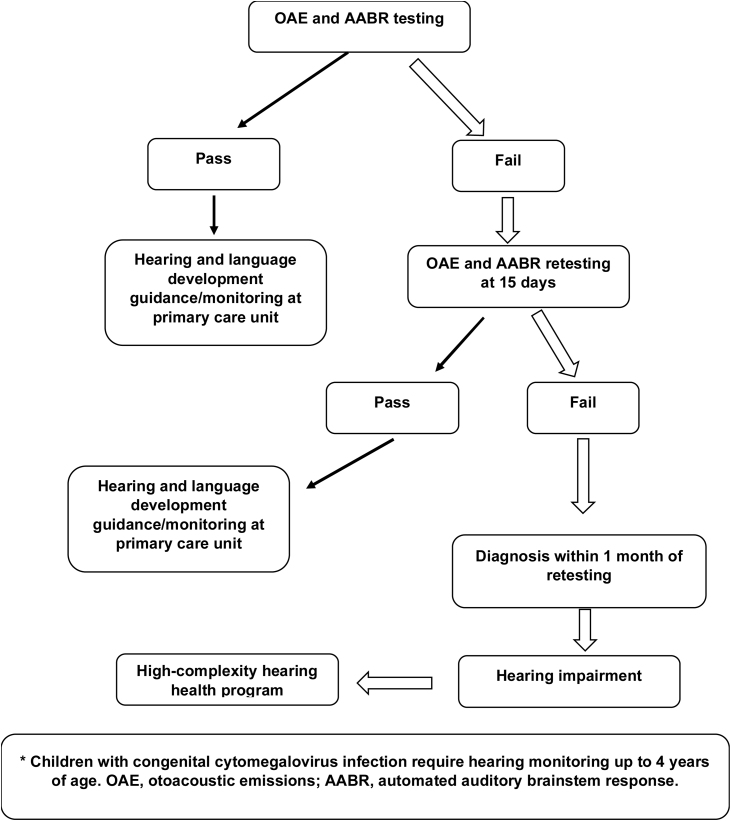
Universal newborn hearing screening flowchart in newborns with risk factors.

Hearing screening of newborns with risk factors (Fig. 1) should consist of both OAE and AABR testing. AABR in this population is essential for identifying auditory neuropathy, which is very common in patients with hyperbilirubinemia, extreme prematurity, and hydrocephalus.^6^ Newborns who pass should be followed up every 6 months for language development at primary health care units. When they fail screening, the test must be repeated up to 15 days and, if they fail again, they should be referred to appropriate centers for diagnosis of the severity and type of hearing loss between 1 month and 3 months of life.

For diagnosis, ABR, automated steady-state response, and distortion-product OAE should be performed. When otitis media with effusion is unclear, 1000 Hz impedance meters should be used in children aged 9 months or older.^6^

If these newborns are hard of hearing, they should be referred for appropriate rehabilitation, which should be started as soon as possible. This can be done through a Hearing Aid (HA) or Cochlear Implant (CI) so that they experience appropriate hearing and speech development.

All children with suspected progressive hearing loss, as in the case of CMV or genetic disorders, require hearing monitoring up to 4 years of age (Fig. 1).

Newborns who are on mechanical ventilation for more than 5 days receive antibiotic therapy for secondary infection prevention, account for 10% to 15% of all live births, and deserve special attention in neonatal hearing screening.^55^ Gentamicin (an ototoxic drug) is used in most cases,^55^ supporting the hypothesis of sensorineural hearing loss in this population. Incubators are potentially noisy and may cause hearing loss.

In the pediatric ICU, newborns have several comorbidities such as malformations, severe infections, and low birth weight. Because they are in a noisy environment (incubators), it is difficult to perform the OAE and AABR tests, which delays hearing screening. The diagnosis is frequently delayed or not made.

Another important factor for not performing the tests in newborns with risk factors is that neonatal ICU speech therapists often do not specialize in audiology. Furthermore, hospitals rarely have audiologists available on holidays or weekends, which may leave these children unattended in case of hospital discharge. A suggestion to solve this problem is to provide training for intensive care nursing staff or to prepare an on-call schedule for audiologists in maternity hospitals.

These children frequently have multiple comorbidities that will be treated after discharge. However, they are often not taken to the office for out-of-hospital hearing screening testing, which causes enormous damage to this population, as they are diagnosed too late, when appropriate auditory rehabilitation is no longer possible.

#### Recommendations

UNHS coverage in Brazil is still very low and has marked regional differences. Measures need to be taken for ensuring nationwide coverage, enabling identification and rehabilitation at an ideal age for children with hearing loss (Strong recommendation – High-quality evidence).

Regardless of UNHS outcomes, all infants and children should be routinely monitored for hearing, cognitive, and oral language development, as well as for achievement of educational milestones (Strong recommendation – High-quality evidence).

Training and hiring human resources to perform in-hospital hearing screening is necessary for ensuring that these tests are done before hospital discharge (Insufficient).

Checking for screening and retesting at the time of first vaccination is a possible strategy for increasing UNHS coverage, with identification of children who may not have undergone hearing screening at the maternity hospital or who did not return for retesting (Insufficient).

Creating a national database with information on tests performed, results, and other data necessary for monitoring and planning actions and adjusting the human, physical, and technological resources for UNHS programs (Insufficient).

### Laboratory testing

Creating a UNHS protocol has multiple benefits, including: Identifying newborns at risk of deafness who could benefit from early intervention but are missed by current screening programs; Providing etiologic information as part of screening; Possibly decreasing the number of children who are lost to follow-up; Potentially saving costs by reducing the frequency of later testing.^21^

Any screening test must weigh false negatives versus false positives to best serve the screened population. “False positive” may refer to the actual screening device used, but here it refers to the overall result given to parents ‒ “pass” or “refer” ‒ versus the subsequent diagnostic confirmatory physiologic testing, which serves as the “gold standard”. The Positive Predictive Value (PPV) is the probability that individuals who screen positive actually have the disease. An ideal screening test would have a high PPV and a high sensitivity. Although negative predictive value and specificity are also considered in the design of screening tests, a confirmatory or diagnostic test should aim for a high negative predictive value and a high specificity as false negatives are reduced.

Once the diagnosis of hearing loss is established, the search for an underlying etiologic diagnosis is recommended. Available guidelines include screening for congenital infections, imaging, and genetic testing.^56^, ^57^ Ophthalmologist consultation has been shown to have a high yield for visual anomalies in children with sensorineural hearing loss.^58^ The investigation may be complemented by a renal ultrasound to check for congenital malformations, an electrocardiogram to rule out long QT syndrome (as seen in Jervell and Lange-Nielsen syndrome), and other tests based on clinical findings. In unilateral hearing loss, the etiologic investigation may be limited to a thorough clinical examination for a syndromic cause of hearing loss, investigation of possible congenital infections, and imaging of the inner ear.^59^

A series of diagnostic laboratory tests that can help determine the cause are shown in Table 6. Since the diagnostic yield of these tests is low (ranging from 0% to 2%),^60^ they should only be ordered if indicated by medical or family history.

**Table 6:** Laboratory tests for evaluation of children with sensorineural hearing loss (modified from Prosser et al.).^61^

Test	Findings	Disease/syndrome
Complete blood count	Presence of blast cells	Leukemia
Blood smear	Sickle red blood cells	Sickle cell anemia
Platelet count	Macrothrombocytopenia	Fechtner syndrome
Antinuclear antibody/antineutrophil cytoplasmic antibody	Increased	Lupus/other autoimmune disorders associated with hearing loss
Erythrocyte sedimentation rate	Increased	Lupus/other autoimmune disorders associated with hearing loss
Rheumatoid factor	Increased	Lupus/other autoimmune disorders associated with hearing loss
PCR	Increased	Autoimmune labyrinthitis
Serum immunoglobulins	Increased	Autoimmune labyrinthitis
C3, C4^LU^	Increased	Autoimmune disorder associated with hearing loss
TSH	Hypothyroidism	Pendred syndrome
Blood urea nitrogen	Increased	Alport syndrome
Creatinine	Increased	Alport syndrome
Urinalysis	Proteinuria	Alport syndrome
Blood glucose	Increased	Alström syndrome/diabetes
FTA-ABS	Positive	Syphilis
Lipid panel	Increased	Dyslipidemia associated with hearing loss
Serum iron	Decreased	Iron deficiency anemia associated with hearing loss
Heavy metals	Increased	Poisoning associated with hearing loss

PCR, Polymerase Chain Reaction; TSH, Thyroid-Stimulating Hormone; FTA-ABS, Fluorescent Treponemal Antibody Absorption.

#### Rubella

Generally, a definitive laboratory diagnosis of congenital rubella infection can only be made within 12 months of birth. Rubella infection may be diagnosed if at least one of the following criteria is met: (1) Positive anti-rubella IgM titer (possibly measured with enzyme immunoassays), (2) Substantial increase in IgG titer 2 to 3 weeks after the acute phase of infection or high titers persisting beyond what can be expected from passive transfer of maternal antibodies, 3) Isolation of rubella virus from cultures of throat, nasal, blood, urine, or Cerebrospinal Fluid (CSF) samples, 4) Polymerase Chain Reaction (PCR) detection of virus in throat swabs, CSF, or surgical specimens (congenital cataract, as the virus can be isolated from the lens).^62^

Additional laboratory test results that can confirm the diagnosis of congenital rubella infection are thrombocytopenia, hyperbilirubinemia, and leukopenia. Although congenital rubella infection has become rare in the developed world since the virus was eradicated from the western hemisphere, cases of imported disease are still observed. Furthermore, congenital rubella infection remains endemic in some low-income countries in the developing world and should thus be considered in the diagnostic workup of hearing loss if infection cannot be excluded for historical or epidemiological reasons.

#### Cytomegalovirus

cCMV has the highest rate of mother-to-child transmission in humans, with a neonatal prevalence ranging from 0.2%‒2%. Symptoms of congenital infection are intrauterine growth retardation, hepatosplenomegaly, petechial skin rash, retinitis, thrombocytopenia, and hepatitis. Although 80%‒90% of newborns do not show symptoms of cCMV, sensorineural deafness may be present at birth, progress in severity, or develop later. cCMV may lead to a wide spectrum of dysfunctions that, in addition to sensorineural hearing loss, include blindness and neurodevelopmental delay.^63^

Foulon et al.^64^ demonstrated a 22% incidence of sensorineural hearing loss in asymptomatic infected individuals. In that study, which followed up children with cCMV for 10 years, the rate of delayed-onset deafness was 5%. However, another study showed that up to 40% of asymptomatic children with congenital infection had sensorineural hearing loss, with a late-onset sensorineural hearing loss rate of up to 50% in this group (mean onset at 44 months).^28^

Sensorineural hearing loss occurred in 9.9% of asymptomatic children and 33% of symptomatic cases according to a systematic review conducted in 2014.^65^ UNHS indicated the diagnosis in only 12.6% of neonates. The authors also reported that a substantial proportion of the children developed hearing loss after the first month of life, while others developed it after the first year of life. There are also sporadic reports of delayed onset, beyond 5 years of age. The incidence of delayed sensorineural hearing loss was 18.1% for symptomatic patients and 9% for asymptomatic patients, accounting for a cumulative incidence of 7%‒11% in studies with more than 5 years of follow-up.^65^

Any newborn with signs of congenital infection should be tested for CMV infection. It is the leading nongenetic cause of congenital hearing loss in high-income countries. This infection should also be considered in children with hearing loss who are healthy and asymptomatic. Other signs and symptoms of CMV infection include microcephaly and jaundice, with sensorineural hearing loss being present in approximately 30% of symptomatic children infected with CMV.^66^ Since the first report in 1964,^67^ this congenital infection has been considered a very expressive cause of sensorineural hearing loss and the most common nonhereditary etiology of congenital sensorineural hearing loss.^67^ Therefore, the development of evidence-based and consensus-based care policies is of interest for public health.

cCMV is a challenge for otolaryngologists and pediatricians. The window period for diagnosis is very short, about 2 to 3 weeks after birth. Available tests become undefined after this period. In addition to this difficulty, there is the fact that most patients are asymptomatic. Low clinical suspicion at birth has consequences for delayed diagnosis and interventions.

Few Brazilian studies have addressed the topic. Yamamoto et al.^53^ conducted a cohort study in Ribeirão Preto (São Paulo, Brazil) evaluating 11,900 neonates, of which 68 (0.6%) were positive for cCMV by viral DNA detection in saliva/urine within the first 3 weeks of life. Ninety-one neonates did not pass UNHS (0.8%), and, of these, 24 (26.4%) had hearing loss confirmed later by diagnostic evaluation; 7 neonates (i.e., one-third of them) were identified with cCMV. Based on the data reported in this Brazilian cohort, the authors emphasized that: Failing the UNHS is twice as common in neonates with cCMV compared with uninfected children, demonstrating that these children may require a diagnostic test for cCMV; Hearing loss is 90 times more frequent in children infected with cCMV compared with uninfected children; One-third of cases of bilateral sensorineural hearing loss and half of cases of unilateral hearing loss are associated with cCMV.

### Congenital cytomegalovirus diagnosis

The diagnosis of cCMV is based on the identification of viral particles in saliva and urine using a PCR technique in the short window period up to the third week of life,^68^ as sensitivity decreases over time. In intrauterine life, after 20 weeks’ gestation, identification can be done through the amniotic fluid. This procedure is indicated in very specific situations.

Although described years ago, this method has not reached consensus. For health funding reasons, testing is most often performed in patients considered a target for the disease, such as symptomatic patients with a suggestive clinical status. If screening for cCMV is performed only in this population, the consensus statements point to a significant proportion of asymptomatic patients and patients with cCMV who would miss the chance of being diagnosed early.^68^, ^69^

According to these consensus statements, in addition to apparent cases, children who fail the UNHS or who present with sensorineural hearing loss of unapparent cause should be tested for cCMV regardless of age.^68^, ^69^ The same applies to neonates born to mothers with a documented seroconversion during pregnancy. According to Cannon et al.,^70^ for cases of cCMV-induced sensorineural hearing loss, there is good evidence that routine newborn screening has a positive impact on communication-related outcomes and should be considered for all children.

Prenatally, a PCR test for CMV in amniotic fluid can confirm cCMV infection (PPV is close to 100%).^66^ After birth, urine, saliva, or throat swabs should be collected and analyzed. Samples must be collected within 3 weeks of birth, as viral shedding after this period may reflect an infection acquired in the postnatal period and thus noncongenital. In children undergoing etiologic evaluation of sensorineural hearing loss over 3 weeks of age, cCMV infection can only be confirmed retrospectively, using stored newborn blood as a model source for PCR-based diagnosis.

In many high-income countries, a blood sample is routinely collected during the first week of life to screen for metabolic, endocrine, and other disorders. If cCMV infection is suspected, in addition to laboratory testing, brain imaging (cranial ultrasound or MRI), visual function assessment, and hearing assessment are required. However, cCMV infection is virtually asymptomatic in 90%of newborns. These children generally have fewer neurodevelopmental problems than those who are symptomatic at birth, but 10% will develop substantial sensorineural hearing loss at some point during childhood.^65^

Because of the cost of implementing universal screening, low- and middle-income countries do not routinely test newborns. In an SBO discussion forum, medical participants from all Brazilian regions consensually encouraged the practice of universal screening for cCMV. If this is not possible, neonates suspected of having this condition should be tested before discharge.

### Hearing evaluation in children with congenital cytomegalovirus: Proposal for an evidence-based protocol

The phenotypes of hearing loss may vary. The most severe forms, characterized as profound bilateral sensorineural hearing loss, seem to occur more often in patients with primary infection, in cases of symptomatic neonates.^64^, ^71^ However, intermediate forms of hearing loss are recognized in long-term follow-up studies.^64^, ^71^ Sensorineural hearing loss in cCMV has been described as unilateral or bilateral, asymmetric, progressive, and fluctuating. Several protocols for longitudinal follow-up of children with cCMV have been described. To propose an evidence-based audiologic clinical follow-up closer to the Brazilian reality, the Ribeirão Preto cohort study^53^ can serve as a basis with small adjustments for each Brazilian region. According to the authors, neonates undergo UNHS at birth, consisting of OAE and AABR testing, to minimize false positives and negatives and to detect sensorineural hearing loss at specific frequencies as well as cases of auditory neuropathy.

Children with cCMV and those who failed the second test, in the case of retesting at 30 days, are referred to specialized centers for diagnostic hearing evaluation until the sixth year of life; assessments are performed every 6 months until the third year, and annually thereafter. The diagnostic evaluation consists of audiometry suitable for the child’s neuropsychomotor development. In the case of free-field audiometry, an OAE test is combined so as not to miss patients with unilateral sensorineural hearing loss. In the presence of any abnormality, immittance testing is performed to rule out the conductive causes of hearing loss so common in children. If a diagnosis is made, children are evaluated by click and tone-burst ABR at specific frequencies to reach a conclusion or classification of sensorineural hearing loss.

### General diagnostic evaluation in children with congenital cytomegalovirus: General guidelines for otolaryngologists and pediatricians

It is important that otolaryngologists and pediatricians are aware of some approaches based on current evidence and consensus statements^68^, ^69^ when conducting a general clinical evaluation in children with cCMV. Patients should undergo imaging assessment of the Central Nervous System (CNS), where transcranial ultrasound is considered the initial test of choice and can be complemented and/or replaced by cranial MRI. As these patients are children, cranial CT should be avoided to prevent high exposure to radiation.^69^

Common findings suggestive of cCMV include CNS malformations, ventriculomegaly, microcephaly, and cortical and periventricular calcifications. Because these changes are present in 80% of patients with sensorineural hearing loss and symptomatic cCMV,^64^ they are relevant for the otolaryngologist providing auditory rehabilitation for these patients. Those with only limited changes have demonstrated the best rehabilitation outcomes.

Other key assessments are hematology, liver enzyme, bilirubin, and renal function tests, especially when pharmacologic therapy is indicated. These children also require a complete ophthalmologic examination for the diagnosis of congenital cataract and chorioretinitis.

### Evidence-based medical treatment of children with congenital cytomegalovirus

Despite the high prevalence and high degree of morbidity, a gold-standard diagnostic test and a definitive treatment for cCMV have not yet been established.^72^ For symptomatic patients, 6-week treatment with valganciclovir has been approved and is associated with improved audiologic levels.^73^

In cases of moderate-to-severe symptomatic cCMV, relevant consensus statements^68^, ^69^ recommend antiviral treatment in the neonatal period for 6 weeks to 6 months. The available drugs are oral and intravenous ganciclovir and valganciclovir. Antivirals as well as hyperimmune gamma globulin, initially suggested as a possible fetal protective measure in mothers with confirmed seroconversion, are not recommended for routine use. The controversy in the literature refers to asymptomatic patients or those with mild forms of cCMV presenting with early or delayed hearing loss.

The recommendations of the European consensus statement^68^ are based on the understanding that these children exhibit an evolutionary situation supposedly linked to the development of the disease in the CNS. Although the real action of the virus in the inner ear remains unclear, the agent is believed to have a possible direct cytopathic effect on the inner ear cells. Conversely, the immune reaction also seems to play a role in the pathogenesis of cCMV-related sensorineural hearing loss.^69^

Although not unanimously, pharmacologic intervention was recommended by most European consensus members for patients with asymptomatic or mild forms of cCMV with sensorineural hearing loss at birth or developing later. However, at a subsequent meeting, the experts did not support this approach because there were few studies showing scientific evidence and most of them were not randomized clinical trials. In the absence of evidence, during the SBO discussion forum, we supported the possibility of pharmacologic intervention with the provision of clear information on the uncertain prognosis and potential harm to the child’s health.

### Evidence-based auditory rehabilitation of children with congenital cytomegalovirus and hearing loss

Since different phenotypes are possible, the choice of auditory rehabilitation method should be individualized and shared with the family of the child with sensorineural hearing loss and cCMV. Most often, a multidisciplinary team is required because different impairments may affect the child’s global development. All forms of auditory rehabilitation can be indicated, and the same patient may need more than one method at different times. Regardless of the method indicated, longitudinal follow-up of the infant with cCMV is important, as it is common for sensorineural hearing loss to progress on the same side or on the opposite side.^74^

Several efforts have been made to establish the prognosis of hearing loss progression in patients with cCMV. The belief that only primary gestational infections generate neonates with symptomatic forms and would be responsible for worse hearing outcomes has not been supported in the scientific literature.^75^

For clinical practice guidance, the following elements are indicative of worse outcomes of sensorineural hearing loss in cCMV and delayed-onset hearing loss^76^: Symptomatic forms of Ccmv; Prolonged period of transmission as detected by urinary viral shedding; Higher viral loads.

Two studies evaluated some predictors of good auditory rehabilitation outcomes for patients with CIs.^77^, ^78^ More advanced white matter lesions on MRI correlated with worse outcomes. However, despite having poorer auditory outcomes in auditory rehabilitation, patients with cCMV, according to these authors, had results that justify the need for the procedure. These variables, considered predictors of auditory rehabilitation outcomes, can be used during the preoperative period in the management of family expectations and counseling.

A more detailed analysis of the methodology of these studies shows that the most important covariate possibly affected is the patient’s intellectual quotient, often associated with the amount of CNS imaging abnormalities, which will certainly exert greater influence on patient outcomes during auditory rehabilitation. Conversely, the anatomic site of the lesions and the presence of changes in the temporal and parietal lobes, sites related to the auditory and motor cortical areas of speech, seem to lead to worse outcomes in the follow-up of children with cCMV undergoing auditory rehabilitation with CIs.

### Evidence-based preventive measures

To date, no vaccines have been approved for marketing in Brazil, but 2 are being tested in the United States in phase II, placebo-controlled, randomized clinical trials. These trials have shown seroconversion in 50% of pregnant women receiving the glycoprotein B vaccine.^79^ In a previous investigation, an important association of seroconversion was reported for 30% of the pregnant women whose children attended day care centers.^80^

The consensus statements recommend that pregnant women and mother-and-child health professionals be clearly informed of the forms of transmission and prevention. The measures aim to protect pregnant women from secretions from children under 2 years of age, especially those attending day care centers as they have a prolonged period of transmission and are subject to constant reinfections. Washing hands after diaper changes, avoiding kissing, and not sharing food and cutlery are measures that have been shown to reduce seroconversion in pregnant women.

Prevention of fetal infection will only be indicated in cases of contamination confirmed by testing via amniotic fluid between 20 and 21 weeks.^74^ Hyperimmune immunoglobulin is indicated for patients admitted to authorized clinical trials. Prevention of vertical transmission will supposedly have an important impact on the occurrence of permanent sequelae, the most frequent being hearing loss.

#### Recommendations

Indiscriminate laboratory testing without diagnostic suspicion is not recommended, as it has a low yield (0% to 2%) (Strong recommendation – Moderate-quality evidence).

Children with sensorineural hearing loss exhibit a 2- to 3-fold increase in the incidence of eye problems, including correctable vision disorders such as astigmatism and refractive errors. Thus, an ophthalmologic evaluation is indicated (Strong recommendation – Low-quality evidence).

Cardiovascular evaluation has a relatively low yield and should be performed if genetic testing or electrocardiography identifies a cardiac abnormality that requires further evaluation. Electrocardiographic changes may be more common among children with unilateral or bilateral sensorineural hearing loss, regardless of the severity of hearing loss (Weak recommendation – Moderate-quality evidence).

cCMV has been a major challenge for several mother-and-child health professionals, and because its most frequent sequela is sensorineural hearing loss, the otolaryngologist should be directly involved in the multidisciplinary team responsible for the diagnosis and rehabilitation process of children (Strong recommendation – Low-quality evidence).

Because of the different phenotypes of sensorineural hearing loss, a complete protocol including follow-up during the first 6 years of life is suggested, as this will cover delayed-onset losses, which are approximately 1% per year in asymptomatic forms and 4% per year in symptomatic forms (Strong recommendation – Low-quality evidence).

Medical treatment with hyperimmune immunoglobulin, as well as antiviral therapy in intrauterine life, to reduce the risk of fetal contamination and sequelae remains under debate, and only well-founded clinical research protocols should be recommended (Insufficient).

Antiviral therapy with the aim of positively impacting the hearing outcome lacks evidence in the literature and is not routinely recommended (Insufficient).

Children with profound sensorineural hearing loss caused by cCMV exhibit impaired CI performance compared with those with a CI but without cCMV. Lower CI performance in children with cCMV can be attributed to comorbidities (Strong recommendation – Moderate-quality evidence).

Currently, there is no vaccine approved for marketing in Brazil, and the educational measures for pregnant women and health professionals regarding prevention of transmission in this population have shown some evidence of efficacy (Insufficient).

The otolaryngologist should participate in all stages of the decision-making process for a child with cCMV and sensorineural hearing loss (Insufficient).

### Genetics

Identifying the cause is important for the prognosis of hearing loss and language development, as well as for the choice of treatment. Genetic testing should be considered in children with hearing loss of unknown cause. It has a high yield, identifying the condition in 44% of patients with bilateral sensorineural hearing loss.^81^ Genetic causes are 2.5 times more common in bilateral sensorineural hearing loss than in unilateral sensorineural hearing loss. The diagnostic yields of genetic testing are 22% and 1% in children with asymmetric or unilateral sensorineural hearing loss, respectively.^81^ Approximately 70% of genetic causes are nonsyndromic.

Genetic deafness can be classified by the type of genetic inheritance: Autosomal recessive inheritance is the most common and has the following profile: sensorineural, severe to profound, prelingual. Classified as DFNB; Autosomal dominant inheritance is most commonly characterized as progressive, post lingual, moderate hearing loss. Classified as DFNA; Mitochondrial inheritance is when a man or woman inherits maternal mitochondrial DNA; X-linked inheritance is when a woman, the carrier of the mutation, transmits the gene that manifests only in male family members.

To date, 124 genes linked to nonsyndromic hearing loss have been identified, 78 of which are autosomal recessive, 51 are autosomal dominant, and 5 are X-linked.^82^ Among these, GJB2 (connexin 26) is the most common. The GJB2 (connexin 26) and GJB6 (connexin 30) genes, both at the DFNB1 locus, are most commonly associated with bilateral sensorineural hearing loss, accounting for up to 31% of genetic cases confirmed in a recent study.

#### Connexin 26

Connexin 26 or GJB2 (Gap Junction Beta-2 protein) was the first gene identified as responsible for nonsyndromic sensorineural hearing loss.^83^ It is located at different sites in the cochlea and in the epidermis. The ion transport ensured by connexin 26 allows the stability of the endolymph and the generation of potential action within the cochlea.^84^ This transport is essential for auditory physiology.

There are over 70 different mutations reported. Heterozygous and biallelic mutations make genetic counseling difficult.^85^ The relatively small size of the entire coding region in the single exon (exon 2) of the GJB2 gene facilitates rapid identification of mutations in this gene.

GJB2 gene mutations account for^86^: More than 50% of sensorineural hearing loss due to autosomal recessive inheritance; 20% of prelingual hearing loss in high-income countries.

One of the most common GJB2 mutations identified worldwide is 35delG^85^ (70% of cases).^87^ In a sequence of 6 guanines, at positions 30 to 35, one of them is missing. The frequency of this mutation as a cause of deafness varies according to ethnicity. If the carriers of this mutation (35delG heterozygotes) are analyzed against the general population, the results will vary. The prevalence is 2.2% in European countries,^88^ 0.6% in the United States, and almost 0% in China, Japan, and Southeast Asia.^87^ In Brazil, it is around 1%.^89^

Other common GJB2 gene mutations in specific populations include: 167delT ‒ Ashkenazi Jews^87^; 235delC ‒ Asians; R143W ‒ Africans.

It is not easy to determine the frequency of 35delG mutation in Brazil because of the heterogeneity of the population. National studies on deaf patients found quite different results for the presence of this mutation. Oliveira et al.^90^ estimated at 22%, and Bernardes et al.^91^ described a 12% frequency of 35delG mutation in the deaf population.

The presence of the heterozygous 35delG mutation, by itself, is not pathogenic, but it can be associated with other compound heterozygous mutations or with the GJB6 gene (DS13S1854 and DS13S1830) and cause hearing loss. In Brazil, the frequency of GJB6 among heterozygotes for the 35delG mutation has varied: 12.5% (Esteves et al.),^92^ and 25% (Piatto et al.).^93^

Batissoco et al.^94^ analyzed patients with congenital deafness and found approximately 50% of genetic origin, with GJB2 and GJB6 mutations being considered an important cause of deafness in Brazil.

#### SLC26A4 gene (pendrin)

This21-exon gene is located on the long arm of chromosome 7. It encodes pendrin, which is a protein expressed in multiple tissues. It is a transmembrane anion exchanger that exchanges mainly Chloride (Cl-), iodide, and bicarbonate (HCO_3_-). It is found in the apical membrane of epithelial cells of the cochlea, endolymphatic sac and duct, and vestibular system, as well as in the thyroid and kidney.^25^

The role of pendrin in the inner ear is to control ion composition and pH by HCO_3_- secretion and Cl- ion reabsorption because of its function as a Cl-/HCO_3_- exchanger. In the kidney, it plays an important role in controlling systemic pH and blood pressure by secreting HCO_3_- and reabsorbing chloride ions. In the thyroid, pendrin controls iodide transport, essential in the synthesis of thyroid hormones.^95^

The SLC26A4 gene accounts for 5% to 10% of cases of hereditary hearing loss. Biallelic SLC26A4 mutations lead to Pendred syndrome or DFNB4 nonsyndromic deafness, both of which are autosomal recessive. Children with Pendred/DFNB4 syndrome are usually born with residual hearing, which is lost at the time of speech acquisition. In these cases, the degree of hearing loss is moderate to profound. Many patients experience sudden hearing loss during adulthood, following head trauma/barotrauma or fluctuating and progressive hearing loss.^96^

In DFNB4, goiter is not present (differing from Pendred syndrome), but there are cochlear malformations, especially EVA. In some populations, up to 80% of patients with EVA have an SLC26A4 gene mutation. Thus, the presence of SLC26A4 should be investigated in all individuals with sensorineural hearing loss and inner ear malformations.^97^

#### Syndromes

Approximately 30% of cases of congenital hearing loss are syndromic and occur with structural or functional anomalies of other organs and systems. Genetic factors are poorly understood, although large genome-wide association studies have identified several new disease-associated loci and massive next-generation sequencing may bring additional knowledge to this field. Table 7 summarizes the main autosomal dominant and recessive syndromes.

**Table 7:** Syndromes with their corresponding genes and phenotypes.

	Gene	Phenotype
**Autosomal dominant syndromes**		
Waardenburg syndrome (WS1)	PAX3	Major diagnostic criteria include dystopia canthorum, congenital hearing loss, heterochromia iridium, pigmentary anomalies of the skin and hair, and an affected first-degree relative. Approximately 60% of affected children have congenital hearing loss; 90% have bilateral loss.
Waardenburg syndrome (WS2)	MITF, other	Major diagnostic criteria are the same as for WS1, except for dystopia canthorum. Approximately 80% of affected children have congenital hearing loss; 90% have bilateral loss.
Branchio-oto-renal syndrome	EYA1	Selected diagnoses include hearing impairment (98%), preauricular pits (85%), branchial anomalies (70%), renal anomalies (40%), and external ear anomalies (30%). Hearing loss can be conductive, sensorineural, or a mixed loss, ranging from mild to profound.
Treacher Collins syndrome	TCOF1, POLR1C, POLR1D	Craniofacial abnormalities with mandibulofacial dysostosis and hearing loss due to ear malformations.
Stickler syndrome	COL2A1	Conductive hearing loss associated with eye abnormalities (myopia, cataract), arthropathy (spondyloepiphyseal dysplasia), and cleft palate.
Neurofibromatosis	NF2	Bilateral vestibular schwannomas, subcapsular cataract, neurofibromas, and other CNS injuries (meningiomas, gliomas).
**Autosomal recessive syndromes**		
Pendred syndrome	SLC26A4	Diagnostic criteria include congenital sensorineural hearing loss that is non-progressive and severe to profound in many cases, but which may be late and progressive; bilateral dilatation of the vestibular aqueduct with or without cochlear hypoplasia; and an abnormal perchlorate discharge test or goiter.
Usher syndrome (USH1)	USH1A, MYO7A, USH1C, CDH23, USH1E, PCDH15, USH1G	Diagnostic criteria include profound, bilateral, congenital hearing loss, vestibular areflexia, and retinitis pigmentosa (commonly not diagnosed until tunnel vision and nyctalopia become severe enough to be noticed).
Usher syndrome (USH2)	USH2A, USH2B, USH2C, other	Diagnostic criteria include mild-to-severe, bilateral, congenital hearing loss and retinitis pigmentosa; hearing loss can be perceived as progressive over time because speech perception declines as decreased vision interferes with subconscious lip reading.
Usher syndrome (USH3)	USH3	Diagnostic criteria include progressive post lingual sensorineural hearing loss, late-onset retinitis pigmentosa, and occasional vestibular dysfunction.
Alport syndrome	COL4A3, COL4A4, COL4A5 (X-linked)	Renal dysfunction (marked by hematuria with progressive renal failure) and ocular changes (with lenticonus and retinal flecks).
Norrie disease	NDP	X-linked vitreoretinal dysplasia associated with microphthalmia, hypoplastic iris, glaucoma, cataract, and blindness, as well as neuropsychomotor delay.
Jervell and Lange-Nielsen syndrome	KCNQ1, KCNE1	Syncope episodes and QT interval prolongation on electrocardiogram.

CNS, Central Nervous System.

Among the autosomal dominant syndromic causes, Neurofibromatosis type 2 (NF2) is characterized by the development of vestibular schwannomas bilaterally, associated with other meningiomas, optic gliomas, ependymomas, and other spinal tumors. The definitive diagnosis involves bilateral vestibular schwannoma (or family history of NF2), plus one of the following: meningioma, glioma, schwannoma, or juvenile posterior subcapsular lenticular opacity and juvenile cortical cataract.^98^ Another cause of hearing loss is branchio-oto-renal syndrome, which involves anomalies of the branchial arches, ears, and renal system. Patients with this syndrome present with preauricular pits, external ear malformations, microtia, narrowing of the external auditory canal, absence of the oval window, facial nerve dehiscence, poor development of the ossicular chain, cochlear dysplasia, EVA, and lateral semicircular canal malformation.^30^, ^98^

Stickler syndrome is a disorder of collagen connective tissue characterized by congenital vitreous anomaly, early-onset myopia, joint hypermobility, and retinal detachment.^98^, ^99^ Waardenburg syndrome affects pigmented cell structures, such as the cochlear stria vascularis, and is the most common cause of syndromic hearing loss.^99^ On CT, it is characterized by EVA, internal auditory canal hypoplasia, decreased modiolus size, and posterior semicircular canal aplasia or hypoplasia.^30^

CHARGE syndrome, characterized by coloboma, heart defects, choanal atresia, growth retardation, genital hypoplasia, and ear abnormalities, is best evaluated by nasal fiberoptic endoscopy and echocardiography to define these malformations. CT scans show superior semicircular canal aplasia, ossicular abnormalities, and vestibular hypoplasia.^30^, ^99^

Treacher Collins syndrome is characterized by mandibulofacial dysostosis.^98^ For all these syndromes, thorough physical examination and complementary tests (such as cranial and temporal bone CT or MRI) are key to diagnosis.

Among autosomal recessive syndromes, Pendred syndrome (further described below) is characterized by sensorineural hearing loss, goiter, and defects in iodine metabolism. Imaging evidence of Mondini dysplasia or EVA as well as laboratory tests demonstrating thyroid dysfunction are required for diagnosis.^30^, ^98^ Jervell and Lange-Nielsen syndrome is characterized by congenital deafness, QT interval prolongation on electrocardiogram, and exercise-induced syncope episodes or anxiety attacks.

Usher syndrome is characterized by sensorineural hearing loss, vestibular dysfunction, and prepubertal-onset retinitis pigmentosa.^98^, ^99^ Complete otoneurologic examination and ophthalmologic examination are useful in the diagnosis. Another syndrome is Refsum disease, characterized by peripheral polyneuropathy, cerebellar ataxia, retinitis pigmentosa, and ichthyosis (extremely dry skin). Complementary tests for diagnosis include CSF analysis (increased protein levels without an increase in the number of cells present in the CSF), 24-h Holter monitoring (as increased incidence of arrhythmias is observed in this group of patients), and measurement of peroxisomal enzyme phytanoyl-CoA hydroxylase (which converts phytanic acid into α-hydroxyphytanic acid).^98^

Other diseases with later auditory manifestations in the pediatric age group include Alport syndrome (with hemorrhagic nephritis, hearing loss, and visual impairment). Diagnosis can be made if 4 of the following diagnostic criteria are met: family history of hematuria, high-frequency progressive sensorineural hearing loss, ocular changes including anterior lenticonus and/or macular flecks, and glomerular basement membrane changes.^98^ Renal function laboratory tests and ophthalmologic examination are imperative. Mitochondrial encephalopathy, lactic acidosis, and stroke-like episodes (MELAS) syndrome (OMIM 540000) presents with normal development in the early stages of life, but progresses with short stature, nausea, migraines, seizures, and alternating hemiparesis, hemianopia, or cortical blindness. Finally, Myoclonic Epilepsy with Ragged-Red Fibers (MERRF) syndrome (OMIM 590060) presents with myoclonic epilepsy, ataxia, dementia, optic atrophy, hearing loss, short stature, and neuropathy.^98^ Tests to evaluate gene mutations are essential for diagnosis.

An etiologic investigation for congenital hearing loss should be performed for many reasons. It allows accurate and personalized counseling, provides relief from guilt in some cases, and assists in hearing rehabilitation. Identifying the etiology may help to choose appropriate therapeutic or management options (e.g., HAs, CIs, or tailored educational needs), may identify coexisting medical problems that need to be treated or monitored (especially when a syndromic genetic cause of hearing loss is found in a child referred as nonsyndromic) and safe preventable risk factors for future hearing deterioration (e.g., aminoglycoside use or head trauma), and may also predict the progression of hearing loss to some extent.^59^

Auditory neuropathy is a common finding in Charcot-Marie-Tooth disease, which is the most common inherited neurologic disorder, affecting 1 in 2500 people and being characterized by progressive motor and sensory neuropathy.^100^ There are several types, and more than 80 genes have been identified; particularly 2 of them, MPZ and PMP22, have been associated with auditory neuropathy. It is an autosomal dominant trait, and affected individuals have normal cochlear hair cells, with marked degeneration of spiral ganglion neurons.^101^

There are other hereditary progressive motor and sensory neuropathies with auditory neuropathy, such as Friedreich ataxia, with possible spiral ganglion degeneration. Cochlear implantation is likely to have suboptimal postoperative results in these individuals, but it is still a treatment option for deafness.^101^

Mohr-Tranebjaerg syndrome (deafness-dystonia syndrome) with optic neuropathy is a progressive genetic neurodegenerative disease characterized by childhood-onset auditory neuropathy, dystonia, and ataxia beginning in the second decade of life, with decreased hearing acuity beginning in the third decade and dementia in the fifth decade of life. Some patients also develop paranoid ideas. It is caused by mutations in the TIMM8A gene, which is an X-linked recessive gene.^102^

### Pendred syndrome

First described in the literature by Pendred in 1896, this syndrome is characterized by sensorineural hearing loss, inner ear malformations (incomplete partition type II and EVA), and thyroid dysfunction.^95^ Pendred syndrome is inherited in an autosomal recessive manner and results from biallelic mutations in the PDS/SLC26A4 gene.^103^ EVA can be observed in nonsyndromic hearing loss if there is homozygosity for wild-type SLC26A4 or only one mutated allele.^104^ Overall, Pendred syndrome has been estimated to account for up to 10% of hereditary hearing loss, with an incidence of 7.5 to 10 in 100,000.^98^

Audiologic phenotypes may vary, with mild-to-profound, congenital or late-onset hearing loss. Most patients present with severe-to-profound progressive congenital hearing loss, which can be aggravated by traumatic brain injury or barotrauma.^105^ Especially in children, the only clue to the diagnosis of Pendred syndrome may be the detection of radiologic abnormalities – EVA and incomplete partition type II. These diagnoses have important implications for management, as children with EVA may present with sudden and significant hearing loss after mild traumatic brain injury.^106^

Goiter is characteristically multinodular, with onset in the second decade of life. It develops during puberty in 40% of cases and in adulthood in 60%.^98^ Thyroid hormones can be at normal (in most cases) or low levels. Delayed thyroid iodine organification results in a positive perchlorate discharge test.^27^

Two other genes have been described to cause Pendred syndrome, accounting for less than 2% of affected individuals: FOXI1 encoding Forkhead box protein I1 and KCNJ10 encoding the adenosine triphosphate-sensitive potassium channel.^27^ Individuals with Pendred syndrome or DFNB4 should undergo annual hearing tests due to the possibility of hearing loss progression. In addition, thyroid function should also be monitored in children with Pendred syndrome, as there is a risk of developing goiter during puberty.^105^

#### Genetic counseling

Comprehensive genetic testing has become more accessible since the advent of Massively Parallel Sequencing (MPS) based platforms that allow the sequencing of billions of DNA base pairs simultaneously, reducing costs and being suitable for large-scale application.^107^ A study of 53,711 US children with sensorineural hearing loss showed that the likelihood of receiving genetic testing increased substantially between 2008 and 2018 (Odds Ratio = 1.22 per year; 95% CI 1.20‒1.24).^108^ However, out-of-pocket costs are still high to some families. Populations with lower socioeconomic status had lower participation in genetic testing.^108^

Patients and families should be informed of the ethical and social issues related to genetic testing, including the psychological impact of the diagnosis, implications for family members, and potential employment or insurance discrimination.^109^ They should also be prepared for the possibility of uncertain findings. Genetic testing may not identify causal mutations or may identify mutations of unknown significance. Furthermore, failure of genetic testing to identify a causal mutation does not exclude the presence of a genetic basis for hearing loss.

Authorization and guidance on genetic testing cannot be overlooked. Genetic testing involves not only the patient but also family members, so it must be extremely confidential. Consent for genetic testing in children should be obtained from both parents and, in the case of a positive result, the otolaryngologist should refer the patient to a geneticist for genetic counseling.

To define uncertain results, follow-up is recommended because characteristics related to certain mutations may only become apparent as the child grows older, at which time additional testing may be indicated.^110^ Follow-up is also recommended because medical advances may contribute with additional genetic testing made available for further causal workup.

#### Recommendations – when to indicate genetic evaluation?

Considering the frequency of genetic causes in the etiology of hearing loss, whenever the diagnosis is uncertain, it should be checked if all other causes have been ruled out. The American College of Medical Genetics and Genomics (2014)^110^ designed a guideline to help clinicians decide when to order genetic testing. First, medical and birth histories should be obtained to help differentiate between acquired versus congenital hearing loss.

For individuals without physical findings suggestive of syndromic deafness and without medical or neonatal history suggestive of acquired causes, nonsyndromic genes should be investigated – single-gene (GJB2) testing using gene panel tests (Weak recommendation – Moderate level of evidence).

Given the high prevalence and costs in Brazil, it is suggested to start investigating the 35delG mutation in GJB2, followed by a gene panel including GJB2 and GJB6 and, if all testing is negative until that moment, large sequencing panels should be used for the different genes involved (Weak recommendation – Moderate level of evidence).

### Radiologic imaging studies

Imaging is extremely important for the diagnostic evaluation of pediatric patients. It plays a role in determining the etiology, identifying abnormalities related to hearing loss progression, and surgical planning.^111^, ^112^ Imaging allows the evaluation of the anatomy of the structures of the cochlea, labyrinth, cranial nerves, and central auditory pathways. In addition, several malformations can interfere with decisions about which hearing rehabilitation methods to use and, in some cases, contraindicate interventions.^113^

The diagnostic yield of imaging studies in the evaluation of children with profound bilateral sensorineural hearing loss ranges from 7% to 74%, with high specificity and high PPV.^114^ The choice of imaging modality should take into account radiation exposure, diagnostic accuracy, duration, need for sedation, and cost.^115^

The imaging modalities of choice for the assessment of pediatric sensorineural hearing loss are high-resolution CT and MRI. CT provides better visualization of bone structures, lower costs, and faster imaging, which makes it easier to use in pediatric patients. However, CT scans expose children to ionizing radiation, and longitudinal studies have shown an increased lifetime risk of developing certain types of cancer.^116^ MRI provides better quality images of soft tissues and neural structures, but it is associated with higher cost and longer acquisition time, often requiring sedation to be accomplished.^117^, ^118^ If there is no suspicion of neoplastic, infectious, or inflammatory processes, intravenous contrast may not be necessary.

Given the several factors mentioned above, there is considerable variation between clinical practice and institutional protocols in diagnostic methods used.^60^, ^119^ Several consensus statements have been published to provide a standardized approach to the assessment of congenital sensorineural hearing loss, but they have not been universally adopted. A systematic review indicates that imaging should play a crucial role in the diagnosis of sensorineural hearing loss in children, with results influenced by both the severity of hearing loss and the results of genetic testing.^120^

Several studies have demonstrated a correlation between diagnostic yield and hearing loss severity, with an increase of almost 20% in diagnostic yield in severe-to-profound hearing loss compared with mild-to-moderate hearing loss. The diagnostic yield is lower in patients who test positive for mutations in the GJB2 gene, which is the most common cause of nonsyndromic hearing loss.^121^

It remains unclear which imaging modality should be ordered, but several studies have reported a higher diagnostic yield with the use of CT than MRI.^122^ It is common to order CT or MRI separately, but several groups have also reported the concomitant use of both methods. Two systematic reviews indicate that CT appears to have a higher diagnostic yield than MRI to diagnose EVA and cochlear abnormalities, whereas MRI can detect more cochlear nerve abnormalities.^123^, ^124^

The diagnostic yield of CT is estimated at approximately 30% based on systematic reviews. This estimate corresponds to a number needed to image of 4, that is, 4 pediatric patients with congenital bilateral sensorineural hearing need to undergo CT to find a morphologic abnormality. In this same systematic review, CT was able to identify 8.7% more findings than MRI.^123^

In a multicenter study evaluating the diagnostic yield of CT versus MRI in children with profound bilateral hearing loss, the concordance rate between CT and MRI was 92%, with the diagnostic yield of CT being 7% higher than that of MRI (*p* = 0.0001).^125^ In another study with a similar design comparing diagnostic yield between CT and MRI, the concordance rate between them was 86%, with MRI having a 14% higher yield for detecting diagnostic findings in children with profound bilateral sensorineural hearing loss.^126^

**Figure 2:**
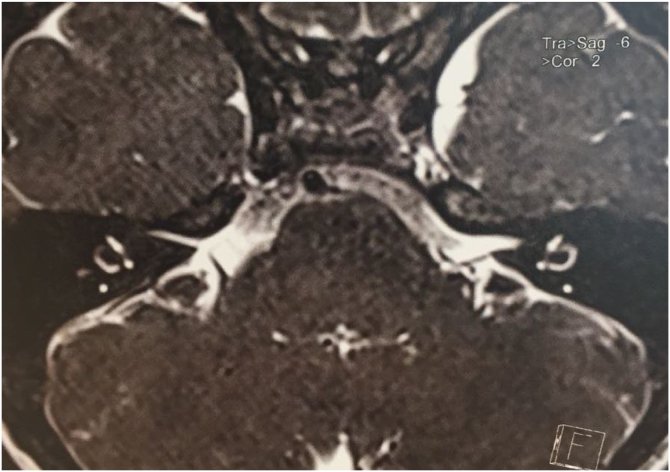
Magnetic resonance imaging (T2-weighted image). Axial plane. Patient with bilateral sensorineural hearing loss. Evidence of eighth nerve aplasia in the right ear.

In cases of auditory neuropathy, MRI proved to be highly effective at detecting cochlear nerve hypoplasia, a common finding in these cases.^127^ Between 12% and 18% of children with congenital sensorineural hearing loss have hypoplasia or aplasia of the eighth cranial nerve (Fig. 2). MRI has the highest diagnostic yield in these cases.^128^ The internal auditory canal may also be hypoplastic in these cases, which can be detected on CT.

The facial nerve has an abnormal course in 16% of children with inner ear malformations, representing increased difficulty in CI surgery, especially when it runs across the promontory or makes the facial recess narrower. In some children, the facial nerve may be split, and a second nerve may be located anteriorly, in addition to a branch located in the normal position of the facial recess.^129^ In these cases, a detailed study with MRI and CT is recommended.

**Figure 3:**
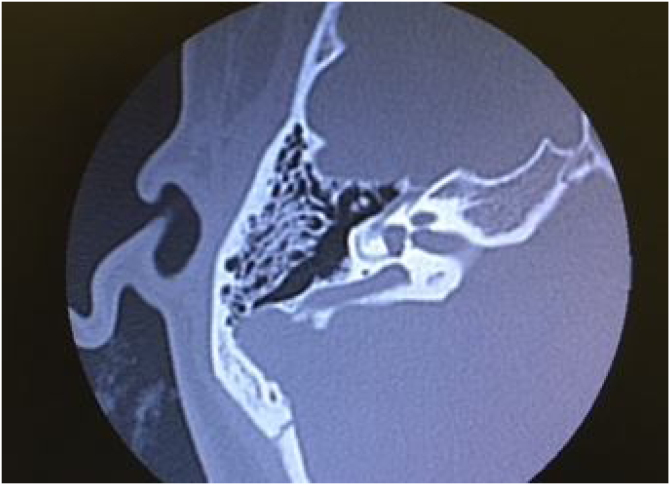
Computed tomography scan of the mastoid. Axial plane. Right ear. Patient with sensorineural hearing loss. Enlarged vestibular aqueduct.

**Figure 4:**
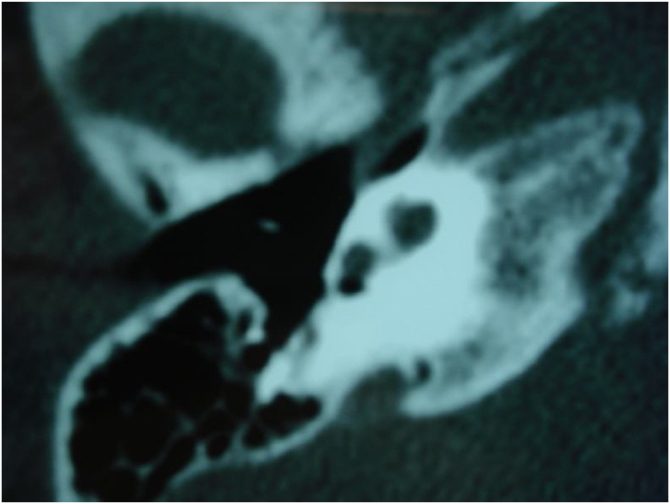
Computed tomography scan of the mastoid. Axial plane. Right ear. Patient with sensorineural hearing loss. Enlarged vestibular aqueduct and incomplete partition type II.

The most common inner ear malformation is EVA, which can be detected by CT or MRI. The modern radiologic definition of EVA has been established as a mean diameter greater than 1.5 mm (Fig. 3).^129^ Mutations in the SLC26A4 gene are a common cause of EVA. It often accompanies other inner ear anomalies, such as incomplete partition type II (Fig. 4). CT is the best imaging modality to assess the vestibular aqueduct. MRI offers complementary visualization of the endolymphatic sac and duct that are usually also enlarged.^96^

**Figure 5:**
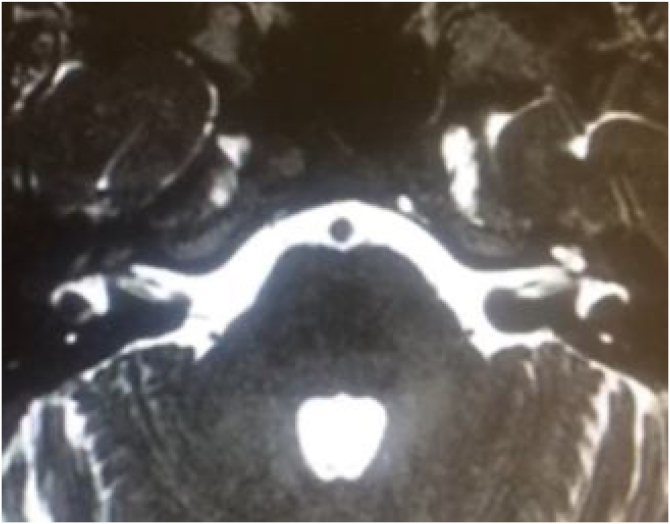
Magnetic resonance imaging (T2-weighted image). Axial plane. Patient with meningitis. Ossification of the cochlea on the right side.

**Figure 6:**
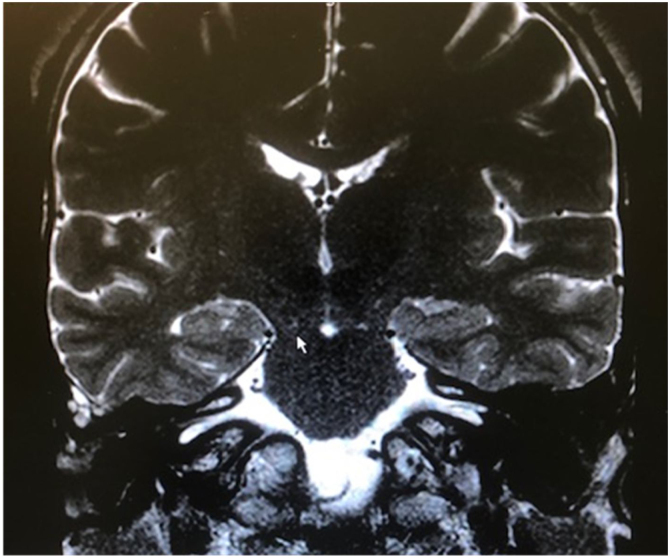
Magnetic resonance imaging (T2-weighted image). Coronal plane. Patient with meningitis. Bilateral ossification of the membranous labyrinth.

Hearing loss after meningitis is the most common cause of acquired bilateral sensorineural hearing loss in the pediatric population. During the acute stage, the CT scan may be normal, but MRI reveals intense cochlear enhancement.^130^ In later stages, fibrosis is shown by loss of fluid signal in the cochlea. Cochlear ossification can be seen on both CT and MRI. MRI is essential to assess the extent of injury and feasibility of CI surgery in these cases (Figure 5, Figure 6).

**Figure 7:**
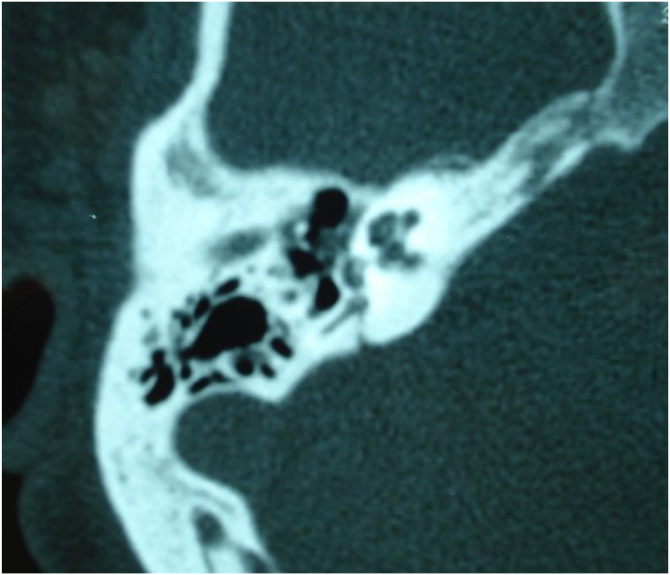
Computed tomography scan of the mastoid. Axial plane. Right ear. Patient with sensorineural hearing loss and peripheral facial paralysis. Presence of transverse fracture with injury to the labyrinth and basal turn that extends to the tympanic portion of the facial nerve.

Traumatic brain injury can lead to drastic conditions, such as facial paralysis and profound sensorineural hearing loss with involvement of the otic capsule (Fig. 7).

**Figure 8:**
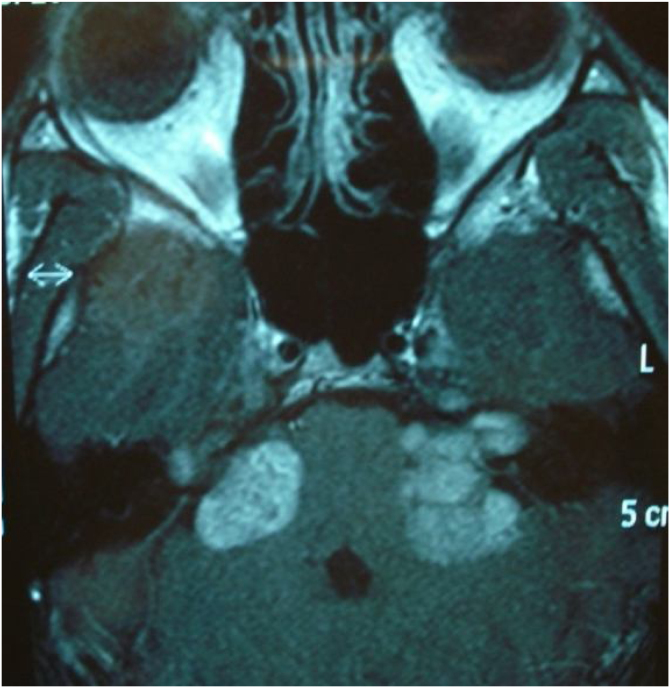
Magnetic resonance imaging (contrast-enhanced T1-weighted image). Axial plane. Presence of bilateral vestibular schwannoma (neurofibromatosis type 2).

Although rare in children, vestibular schwannomas can occur bilaterally (NF2) (Fig. 8). MRI is the most suitable imaging technique in these cases.

#### Recommendations

Imaging is essential during the etiologic evaluation of children with bilateral sensorineural hearing loss due to its high diagnostic yield (Strong recommendation – High-quality evidence).

The choice of imaging modality should consider diagnostic yield, radiation exposure, need for sedation, and cost, but mainly the availability of each modality in each region (Insufficient).

The MRI scan, in particular heavily weighted T2 sequences (CISS, SPACE, FIESTA), offer most of the information about cochlear nerve size and integrity, unusual morphologic inner ear anatomy, and intracranial anomalies that might identify the etiology of deafness and possibly affects the cochlear implant process. (Strong recommendation – High-quality evidence).

Temporal bone CT scan should be considered for children with trauma of temporal bone, craniofacial, and cochlear anomalies at risk of having more surgically challenging anatomy in cochlear implant. (Strong recommendation – Moderate-quality evidence).

Conductive hearing loss ‒ temporal bone CT scan is the preferred exam. (Strong recommendation – Moderate-quality evidence).

### Vestibular assessment of children with hearing impairment – when to indicate?

In humans, the regulation of the body balance occurs through the integration of multiple systems and organs that send information to the CNS (classically, the brainstem and cerebellum), process this information, and send signals and reflexes to control our posture and gait. Among the different sensory systems that contribute to maintaining balance, the following stand out: vision, vestibular system, and proprioception (a bundle of nerves, muscles, and joints).^131^

The vestibular system consists of 3 semicircular canals (anterior, posterior, and lateral) and 2 otolith organs (saccule and utricle). Each of these 5 structures has sensory receptors consisting of highly specialized hair cells, with the ampullary cristae located in the ampullae of the semicircular canals, and the utricular and saccular maculae located in the corresponding otolith organs.^132^ The ampullary cristae are responsible for detecting angular accelerations, such as moving the head in different directions, whereas the maculae are responsible for detecting linear accelerations and head tilt, both in the vertical plane (saccular macula) and in the horizontal plane and in inclination (utricular macula).^133^

The vestibular system has a close anatomic relationship with the cochlea, sharing the same bone scaffold and membranous ducts, having similar sensory hair cells, and sharing the same embryonic origin. Thus, disturbances in one system can affect the other system and vice versa.^134^ Several studies have demonstrated a close relationship and high prevalence of vestibular disorders in children with congenital sensorineural hearing loss, ranging from 20% to 70%. Some studies even suggest a relationship between the etiology and severity of hearing loss and vestibular disorders. Children with severe-to-profound bilateral hearing loss would be more likely to have vestibular dysfunction, and some etiologies, such as cCMV infection and GJB2 gene mutations, would also be more closely related to vestibular deficits.^135^

Unlike hearing disorders, which are more easily diagnosed using different methods, vestibular dysfunction is rarely confirmed in early childhood, and, in most cases, it is not even considered by the physicians who first assess the child, such as pediatricians and otolaryngologists. Perhaps the main reason for this is a lack of knowledge of the importance and function of the vestibular system and/or the unavailability of diagnostic methods for pediatric vestibular assessment.^134^, ^135^

It is important to note that vestibular dysfunction in childhood presents as a delay in some motor development milestones, such as neck control, sitting, crawling, and walking. This makes some children more susceptible to falls and deficits in fine motor skills, thus leading them to avoid, often unconsciously, exposure to more challenging situations involving body balance, such as walking on unstable surfaces (e.g., soft sand). Clear signs, such as the presence of spontaneous nystagmus, are not so common because most patients have bilateral vestibular dysfunction.^134^, ^135^, ^136^

There is no doubt that major challenges of vestibular assessment in children with hearing impairment include the methods available for assessing labyrinthine function, their limitations, parameters, and particularities to be performed in this pediatric population.

#### Vestibular function assessment methods

Among the various clinically available vestibular assessment methods, it is important to emphasize that all, absolutely all, have some limitations. As mentioned earlier, there are 5 sensory organs in the labyrinth (3 cristae and 2 maculae), and no method can assess all organs at once. Another major limitation of tests of labyrinthine function is the indirect assessment of the vestibular system through reflexes, analyzing both the vestibulo-ocular and vestibulospinal reflexes. That is, they evaluate reflex responses generated by the vestibular system, after a stimulus, and not the labyrinth directly. Another extremely important factor to be considered is that vestibular tests are essentially functional tests and not diagnostic tests. The available tests that are best suited for the assessment of the pediatric population are as follows:

• Clinical Head Impulse Test (cHIT): This test was first described in 1988 by Australian professors Michael Halmagyi and Ian Curthois, and to this day it remains one of the most effective bedside tests to assess vestibular function, although not quantitatively. The test is usually performed with the child sitting on the parents’ lap. While standing in front of the child, the clinician holds the child’s head with both hands and asks the child to maintain gaze on the examiner’s nose. Once a steady gaze has been achieved, the clinician rotates the child’s head with a fast, low-amplitude, high-acceleration head impulse to both sides randomly. Patients with a normal Vestibulo-Ocular Reflex (VOR) can often keep their eyes on the target even under rapid head movement, whereas patients with reduced VOR gains often perform corrective saccades to keep their eyes on the target.^137^ Although it is a simple test with high specificity that can be easily performed by any minimally trained health professional, there are some disadvantages: it has an approximately 50% sensitivity, is examiner dependent, and provides only qualitative (not quantitative) information; that is, it does not accurately measure vestibular function. Nevertheless, it is an excellent bedside test that clinicians have at their disposal to assess vestibular function in pediatric patients with hearing loss.^138^
• Video Head Impulse Test (vHIT): This test was introduced in the routine practice of vestibular assessment in the last decade. It is an evolution of the bedside cHIT. The vHIT is the only commercially available vestibular assessment method that can evaluate VOR gain in the 3 semicircular canals in the high frequency spectrum, in addition to being able to quantify the level of residual vestibular function. It also has several other advantages over traditional vestibular tests. It is a rapid, highly reproducible, low-cost test that is not uncomfortable for patients.^137^ This test uses a pupil detection technology with high-precision cameras and an accelerometer, which, through a mathematical algorithm, can correlate eye movement with head movement. The 3 most popular commercially available vHIT brands are EyeSeeCam (Interacoustics, Denmark), ICS Impulse (Natus, USA), and Ulmer (Synapsys, France). However, they differ from each other. Both the EyeSeeCam and ICS Impulse use goggles mounted on the patient’s face, whereas the Ulmer system does not. This is extremely important for pediatric assessments. The goggle placement is very limited in children under 2 years of age. There are no models available with goggles that fit on young children’s faces. In older children, the goggles fit perfectly on their faces and the test is very well tolerated by this population. Perhaps, among all vestibular assessment methods, vHIT is currently the most popular test, even with some limitations.^139^
• Vestibular Evoked Myogenic Potentials (VEMP): There are 2 types of VEMPs, cervical VEMP (cVEMP) and ocular VEMP (oVEMP). These tests assess the function of otolith organs (saccule and utricle, respectively). The tests are of vestibular origin, and measurements are most commonly made in the sternocleidomastoid muscle (cVEMP) and in the inferior oblique extraocular muscle (oVEMP). To attain optimal test quality, the patient must contract the muscles measured, which poses a slightly limiting factor in the pediatric population. Child cooperation is mandatory and necessary for a few minutes. The cVEMP can be more easily performed, with electrodes being placed on the sternocleidomastoid muscle, forehead, and suprasternal notch. Because the test requires child cooperation to be performed, most authors consider the cVEMP abnormal if there is no response in at least 3 consecutive attempts. Like vHIT, cVEMP is often used for vestibular assessment in children with hearing loss, and it can be performed successfully in most cases. It is worth mentioning that cVEMP is better tolerated than oVEMP in this specific population.^138^
• Caloric test: For many years, this test was considered the gold standard for the assessment of vestibular function. It was first described by Robert Bárány, a Hungarian physician, in the early 20th century. It mainly assesses the function of the lateral semicircular canal in the low frequency spectrum through a nonphysiologic stimulus (irrigation at different temperatures). The caloric test is often performed using a traditional protocol with 4 irrigations at different temperatures in both ears. Traditionally, it is a longer test and somewhat uncomfortable for most patients.^140^ Because of technical difficulties associated with the caloric test, other protocols have emerged in an attempt to adapt it to the pediatric population. One of the most commonly used is the mini-ice-water caloric test. In this protocol, the child is kept in a dark environment under video-oculoscopy and placed in the lateral decubitus position with the test ear facing upward. The child’s external ear canal is filled up with 6°‒10 °C water for 10 s. The child is then turned to the prone position, and the eyes are expected to show a nystagmus toward the untested ear lasting for 90 to 120 s for a normal vestibular function.^138^

In general, most authors agree that performing vestibular tests in children is challenging, although approximately 50% of the children undergoing such tests are able to successfully complete a test battery.^141^ Among the various vestibular tests, cHIT and vHIT are the best tolerated by children (65.7% to 94.2%).^138^ Therefore, it is suggested that cHIT be performed as a type of vestibular function screening in the pediatric population, as it is the best tolerated test, also considering that, despite its low sensitivity, a normal response is indicative of a likely normal VOR gain at high frequencies. In cases of uncertain cHIT results, or in the presence of a clearly abnormal response, the child may undergo other tests, with the vHIT, cVEMP and caloric test being the most recommended ones (in this order).^139^

#### Cochlear implant and vestibular dysfunction in children

Currently, the importance of CI surgery for hearing rehabilitation in children with severe-to-profound bilateral sensorineural hearing loss is unquestionable. This method has advanced in the last 2 decades not only in implant technologies, with increasingly less traumatic electrodes, but also in surgical techniques, which have become minimally invasive, following the concepts of soft surgery. While it is known that CIs can restore hearing, it has also been confirmed histopathologically that CIs can damage several vestibular organs, resulting in poor vestibular function, dizziness, and balance disorders.^142^

Most studies aiming to investigate vestibular function in patients before and after CI surgery have been conducted in the adult population, precisely because of the technical difficulties associated with performing vestibular tests in children, as described above. There is still a lack of studies investigating this issue in an exclusively pediatric population. Therefore, the functional and anatomic consequences of CI surgery for children’s vestibular function remains unclear.

The advances in CI surgery have led to an increase in sequential bilateral cochlear implantation in children due to the clear advantages of this technique concerning auditory outcomes, such as better development of the auditory cortex, improved sound localization, and significantly better speech production. However, there has been an increased concern about the potential bilateral vestibular consequences that this technique might induce.^143^ Some studies have reported an incidence of vertigo after cochlear implantation in adults ranging from 2% to 35%, whereas vestibular dysfunction ranges from 20% to 80% after cochlear implantation, depending on the method used to assess labyrinthine function.^144^

Pathological studies performed in adults after cochlear implantation have provided increased evidence of endolymphatic hydrops and saccular collapse. Probably for this reason, the cVEMP test appears to be statistically significantly altered in some studies when comparing results before and after surgery.^142^, ^144^ Interestingly, vHIT results before and after surgery have not shown the same abnormality rates of cVEMP and caloric test results, suggesting that the otolith organs are more vulnerable to damage caused by the surgical procedure than the semicircular canals, a finding compatible with the results of pathological studies, and that high-frequency labyrinthine analysis methods are less sensitive than low-frequency methods for postoperative analysis.^144^

#### Recommendations

There is a relationship between sensorineural hearing loss and vestibular dysfunction in pediatric patients (Strong recommendation – Moderate-quality evidence).

There is methodological difficulty in performing vestibular tests in children, as well as in the parameters of normality for such tests in this specific population (Insufficient).

Among the various tests, cHIT and vHIT were the best tolerated by pediatric patients (Weak recommendation – Moderate-quality evidence).

Despite the difficulties in performing vestibular tests, whenever possible, they should be ordered for children who will undergo CI surgery, both unilaterally and in simultaneous or sequential bilateral cases (Strong recommendation – Low-quality evidence).

Children undergoing CI surgery experienced vestibular deterioration postoperatively, especially in cVEMP and caloric test, suggesting that the otolith organs are more affected and that low-frequency tests are more sensitive than high-frequency tests for the analysis of the impact of cochlear implantation on the vestibular system (Strong recommendation – Low-quality evidence).

### Auditory neuropathy spectrum disorder

Auditory Neuropathy Spectrum Disorder (ANSD) is a common cause of hearing loss, affecting 1.2% to 8.4% of those with hearing loss depending on the population.^145^, ^146^ Auditory neuropathy is a term used to describe individuals who have hearing loss with normal function of cochlear hair cells. Hearing loss is characterized by OAEs and/or presence of cochlear microphonics, indicating normal cochlear function, and by altered ABRs measured by AABR testing, indicating abnormal transmission of the synaptic signal to the CNS.^146^, ^147^, ^148^

ANSD often encompasses auditory dyssynchrony and auditory neuropathy, which, in some cases, cannot be distinguished from each other. The site of the injury that causes auditory neuropathy may involve: (1) Presynaptic region, where glutamate is released by hair cells; (2) Synapse, involving postsynaptic excitatory neurotransmitters; (3) Region of onset of postsynaptic excitation (Excitatory Postsynaptic Potential [EPSP]) on the terminal dendrite; (4) Region along the spiral ganglion that affects neural signal transmission from the auditory nerve to the brainstem.

In cases of auditory neuropathy, inner and outer hair cells, regardless of neural signal transmission, have injuries that result in auditory dyssynchrony. Therefore, individuals typically have deficits in temporal processing of sound that result in impaired speech perception and sound localization.

ANSD is caused by both genetic and environmental factors. There are 13 known genes. Environmental causes primarily affect newborns and include hyperbilirubinemia, thiamine deficiency, and hypoxia. ANSD can also be noise-induced or age-related. Spiral ganglion neurons are bipolar and have a cell body located in the cochlear modiolus, peripheral axons distributed along the spiral limbus toward the row of cochlear inner hair cells, and proximal axons that synapse in the midbrain. These neurons are tonotopically organized with the cochlea.^149^

Multiple spiral ganglion neuron fibers synapse with each inner hair cell. Their axons are myelinated and project to the brainstem. In response to glutamatergic stimulation at the synapse, EPSPs in the peripheral axon lead to gradual Na+ influx via a large number of channels, which allows temporal encoding of acoustic stimuli.^149^, ^150^

Genetic lesions that affect the transmission of the auditory signal to the brain lead to a loss of temporal precision and dyssynchrony, with a degraded potential and altered perception and/or difficulty understanding sound, thus leading to deafness due to auditory neuropathy.

#### Synaptopathies

Synaptopathies can be classified specifically based on the site of synapse injury as presynaptic or postsynaptic. Auditory synaptopathies can be distinguished from auditory neuropathies by electrocochleography. Individuals with synaptopathy appear to have improved adaptation to frequency specific tones, whereas those with neuropathy have low frequency adaptation.^151^

Calcium influx at the base of the inner hair cell near the synapse is mediated via the Cav1.3L-type calcium channel, encoded by the CACNA1D gene. Otoferlin, encoded by the OTOF gene, is a protein responsible for regulating the exocytosis of glutamatergic vesicles in the presynaptic region. The vesicular glutamate transporter type 3 (VGLUT3 gene) is responsible for the glutamate uptake at the postsynaptic site.^152^, ^153^

#### Spiral ganglion and auditory nerve

Mutations in the AIFM1 gene cause neuropathy and are associated with Cowchock syndrome, a neuromuscular disorder associated with deafness and cognitive impairment. It is an X-linked mutation where, in addition to the initial auditory neuropathy, individuals later develop sensory neuropathy with extremity numbness, unsteadiness, and areflexia. It is rare, and to date, there are only 11 reports of AIFM1 mutations causing deafness.^151^

The AIFM1 gene encodes apoptosis-inducing factor 1, a flavoprotein located in the mitochondrial intermembrane space within the inner and outer hair cells as well as spiral ganglion neurons. The AIFM1 protein plays a role in oxidative phosphorylation by controlling the production of intracellular free radicals. Some patients with these mutations have cochlear nerve hypoplasia. To date, there are no reports of cochlear implantation in these individuals, but they are expected to have poor outcomes, particularly those with cochlear nerve deficiency.^150^

Mutations in the NARS2 gene are autosomal recessive and cause nonsyndromic auditory neuropathy as well as Leigh syndrome, an early-onset progressive neurodegenerative disorder affecting the CNS. The NARS2 gene encodes the mitochondrial asparagine-tRNA ligase protein. The NARS2 protein is expressed in the spiral ganglion and other cells of the organ of Corti.^154^

#### Genes with effects on the synapse or spiral ganglion

Mutations in the TMPRSS3 gene cause 2 types of autosomal recessive sensorineural hearing loss: severe-to-profound congenital deafness (DFNB10) and progressive postlingual deafness (DFNB8). There are reports of 41 TMPRSS3 mutations identified as causing deafness. This gene encodes the transmembrane serine protease 3 protein, expressed in inner and outer hair cells and in the spiral ganglion.^155^

Mutations in the TBC1D24 gene cause hearing loss, genetic epilepsy, onychodystrophy, mental retardation, and seizures. TBC1D24 mutations cause severe-to-profound congenital autosomal recessive nonsyndromic sensorineural hearing loss, in addition to progressive dominant nonsyndromic sensorineural hearing loss. The TBC1D24 gene encodes the Tre2-Bub2-Cdc16 protein expressed in the cilia of inner and outer hair cells and in the spiral ganglion. TBC1D24 mutations cause postsynaptic auditory synaptopathy.^150^

Mutations in the DFNB59 gene are the second genetic cause of auditory neuropathy. The protein encoded by DFNB59 is important for the formation of peroxisomes that protect cells from damage during cellular antioxidant response. It is expressed in outer and inner hair cells and spiral ganglion neurons. To date, there are reports of 16 deafness-causing mutations in this gene, 2 of which are related to auditory neuropathy. There are no reports of CI outcomes in individuals with deafness caused by DFNB59 mutations.^102^, ^150^, ^156^

### Otolaryngologic evaluation in children with suspected autism spectrum disorder

The reported incidence of Autism Spectrum Disorder (ASD) has increased dramatically over the past 2 decades, and these trends have led to an increase in the number of referrals to the otolaryngologist for evaluation of individuals with ASD.

According to the Diagnostic and Statistical Manual of Mental Disorders, Fifth Edition (DSM-5), ASD is characterized as a neurodevelopmental disorder marked by impairment in social interaction, communication, and repetitive/restrictive behaviors.^157^ Moreover, individuals with ASD may present with cognitive disorders of attention and memory, lack of eye contact, affection and reciprocal interaction, in addition to impaired language development.^157^

Autism remains a complex diagnosis because of its phenotypic heterogeneity, and its etiology is still debated. Some studies have shown a series of factors involved in the etiology that include genetic, immunological,^158^, ^159^ and environmental aspects, such as prematurity, viral infections, and exposure to agrochemicals,^160^ which may also be present in combination.^158^, ^159^

The pioneering study on the epidemiology of autism was conducted by Lotter, who reported an incidence of 4.5 diagnosed children per 10,000 in England.^161^ In the United States, the Centers for Disease Control and Prevention (CDC) have implemented a surveillance system to estimate the incidence of ASDs in 11 US states, with the first report published in 2000 resulting in 1 in every 150 children being autistic.^162^ In the most recent report, the results point to an incidence of 1 in every 44 children diagnosed with ASD.^163^

In Brazil, there is a lack of studies addressing the epidemiology of ASD nationwide. A study in the state of Santa Catarina found a prevalence of autism of 1.31 per 10,000 population.^164^ In a study conducted in southern Brazil, the prevalence of autism was 3.85 per 10,000 population.^165^ Another Brazilian study, conducted in the Federal District, reported a substantial increase in the rate of schoolchildren diagnosed with ASD and pervasive developmental disorder compared with other disabilities and disorders between 2012 and 2017.^166^

The increase in the diagnosis of children with ASD, as reported in the literature,^163^, ^167^ may be explained by greater dissemination of knowledge, modification and broadening of diagnostic criteria, increased availability of services, and the actual increase in children born with the disorder.^168^, ^169^

Some warning signs to identify children with ASD are also common to children with hearing loss, such as not looking when called and language impairment. Therefore, since hearing loss may be the cause of language impairment, or it may even co-occur with the diagnosis of ASD,^170^ audiologic assessment should be part of the test battery for children with speech delay and under investigation for ASD. It is important that this assessment be performed as soon as possible, since early diagnosis favors both ASD intervention and auditory development, providing multidisciplinary support.^171^

In the literature, there is evidence of abnormal otolaryngologic and audiologic findings in objective and subjective^172^ tests in individuals with ASD, even in the absence of hearing loss, including changes in ABR absolute and interpeak latencies,^173^, ^174^, ^175^ reduced amplitude in cognitive auditory event-related potential (P300),^176^ changes in central auditory processing,^177^ behaviors interfering with the measurement of auditory thresholds in pure tone assessment,^172^ and auditory hyper-responsiveness.^170^

Assessing the presence of hearing loss or impairment in individuals with ASD can be an even greater challenge in young children. In this age group, the otolaryngologic and audiologic evaluation involves a battery of physical examination and behavioral, electroacoustic, and electrophysiologic assessments guided by the crosscheck principle, in which possible differential diagnoses are analyzed before the audiologic diagnosis is accepted.^178^

OAE, immittance, and ABR testing, which are objective tests commonly recommended and used in clinical otolaryngologic practice, require the child to remain motionless, to wear headphones and have a probe inserted into the external auditory canal, and to have electrodes placed in the head and ears, which often makes assessment difficult in children with ASD.^170^

Therefore, objective assessments should be performed during natural sleep, and it is often necessary to schedule one or more visits to complete them; in some cases, they need to be performed with sedation in the operating room. Additionally, in subjective assessments, the child may have interfering behaviors, such as not conditioning to the test, not cooperating with the examiner, paying poor attention to the task, engaging in excessive activity, not tolerating headphones, and being combative toward the examiner.

Therefore, otolaryngologic and audiologic assessments in these children may be a difficult task for health professionals and families. It is important to investigate these difficulties in order to propose solutions, guidelines, and improvements in the environment, procedure, and evaluation protocol, minimizing assessment time and the number of sessions and favoring a timely diagnosis. Moreover, identifying the adversities of audiologic assessment in ASD allows the health professional to interpret the test findings critically, thus helping to rule out or confirm a diagnosis of hearing loss, in addition to making it possible to recognize ASD diagnostic biomarkers.^179^

#### Epidemiology of autism spectrum disorder

Lotter (1996)^161^: Incidence of 4.5 diagnosed children per 10,000 (England).

United States (CDC), incidence in 11 US states: 2000* 1 in every 150 children; 2021* 1 in every 44 children.

Ferreira (2008)^164^: Prevalence of 1.31 per 10,000 (Santa Catarina, Brazil).

Beck (2017)^165^: Prevalence of 3.85 per 10,000 (southern Brazil).

Alves (2018)^166^: Substantial increase in the rate of schoolchildren diagnosed with ASD and pervasive developmental disorder compared with other disabilities and disorders between 2012 and 2017 (Brazilian Federal District).

Protocol for otolaryngologic and audiologic evaluation of children with suspected and/or confirmed autism spectrum disorder in the perception of parents and/or caregivers

### Objectives

To identify factors that can complicate otolaryngologic assessment in ASD.

To identify the main hearing tests and evaluation protocols used in this population.

To identify the main child behaviors that can interfere with assessment.

To characterize the group of children with suspected and/or confirmed ASD who have undergone hearing assessment.

Protocol for otolaryngologic evaluation of patients with a presumptive diagnosis of autism spectrum disorder (address all steps)

Call center/reception: Proper training of call center staff or care team.

Call center/reception: Guidance on conducting the assessments.

Child must be asleep during some of the audiologic tests (sleep in the center).

Test should be scheduled following the child’s usual bedtime routine.

Sleep deprivation; bring the child tired (playing or other activities).

Does the child tolerate headphones? (Assess need for desensitization).

Is the child afraid of physicians in white coats?

Family – Special support from family and friends.

Otolaryngologists – Acknowledge the need for different approaches to patients and their families.

Audiologists – Acknowledge the need for different approaches to patients and their families.

Child must be asleep during electrophysiologic and electroacoustic assessments.

Crosscheck principle.

Outpatient: Perform meatoscopy; explain the need for a complete hearing assessment and suggest a previous assessment with an otolaryngologist; record behaviors identified during audiometry.

#### Clinical evaluation

Because of the high prevalence of ASD in children with visual impairment or deafness, they should be evaluated for ASD in the presence of visual or hearing impairment.

Visual and auditory rehabilitation should be started early, regardless of the degree of impairment; however, success with these devices may vary.

Family should always receive guidance, and it is important that patients and family members have real expectations.

Hearing loss may be the cause of language impairment, or it may even co-occur with the diagnosis of ASD.

#### Genetic assessment

Genetic testing in individuals with autism is far from being routinely performed and does not always yield practical results, but the information provided can change the diagnosis and, consequently, the treatment. None of the molecules or syndromes currently linked to ASD has been proven to selectively cause autism. It appears to result in a variety of abnormal neurobehavioral phenotypes, including ASD and nonsyndromic mental retardation. The identification of molecular links between different ASD-related syndromes will lead to the identification of major deregulated signaling pathways in ASD.

### Associated syndromes

Selected genetic syndromes that are known etiologies of ASD: 22q11.2 deletion syndrome, including velo-cardio-facial syndrome (Shprintzen); Angelman syndrome; CHARGE syndrome; Cornelia de Lange syndrome; Fragile X syndrome; Mutations in *MED12* (Lujan-Fryns syndrome); Prader-Willi syndrome; Mutations in PTEN ‒ Phosphatase and Tensin (Cowden syndrome and Bannayan-Riley-Ruvalcaba syndrome); Rett syndrome; Smith-Lemli-Opitz syndrome; Smith-Magenis syndrome; Sotos syndrome; and Tuberous sclerosis.

Management of patients with suspected autism spectrum disorder in the office

### Inpatient

  • Otoscopy (conductive component).
  • Medical team orders the complete auditory test battery: Audiometry or assessment of auditory behavior; Transient-evoked and distortion-product OAE; Immittance testing or impedance audiometry; ABR; Steady-state response; and Tone-burst ABR.

### Outpatient

Has an otolaryngologist seen the patient?

Scheduling team (call center/reception).

Tests require specific preparation. An otolaryngologist should see the patient because, if there is any impediment, the tests will not be performed.

#### Recommendations

A number of factors should be considered in the otolaryngologic evaluation of patients with suspected ASD:

Ensure an audiologic diagnosis and early rehabilitation before proceeding with any further investigation (Insufficient).

Indicate early language stimulation (Strong recommendation – High-quality evidence).

Evaluate the constantly expanding and evolving list of available laboratory tests in light of the published literature (Weak recommendation – High-quality evidence).

Recognize the phenotypes of well-described syndromic and metabolic conditions that overlap with ASDs (Weak recommendation – High-quality evidence).

Define an individualized assessment plan based on medical history and clinical features (Insufficient).

### Noise-induced hearing loss in childhood and adolescence

The WHO warns that 1.1 billion young people are at risk of hearing loss due to prolonged and excessive exposure to loud sounds.^39^ Children, adolescents and young adults are at higher risk of developing hearing loss because of frequent exposure to loud music during leisure activities, on transport services, and while playing sports.^40^, ^41^ Headphones improve the listening experience but also increase the risk of hearing loss, which is irreversible and manifests initially at 3, 4, and 6 kHz, extending to adjacent frequencies as it progresses.^180^

The use of portable music players such as MP3 players, iPods, smartphones, and similar devices has increased worldwide over the past few decades. They can produce sound pressure levels of up to 126 dBA.^181^ Most studies on noise-induced hearing loss began to be conducted in the 1950s and 1960s in the workplace and in the military. Recreational noise exposure is a recent concern. Exposure to portable music players has been shown to be associated with the development of noise-induced hearing loss and tinnitus in adolescents and young adults. The use of these devices has been associated with a 70% increased risk of mild-to-moderate hearing loss.^182^ Nightclubs and concerts are other common sources of noise, ranging from 104.3 to 112.4 dB HL, with an average level of 97.9 dB HL.^183^

In fitness sports, the use of loud music is widespread. A study conducted at an aerobics endurance tournament showed music intensity levels ranging from 101 to 119 dB during 120 min of activity.^183^ High-intensity music in gym classes has been shown to be related to enjoyment and motivation to work out. Exercisers generally do not consider it dangerously loud. Aerobic activities and their variants become high-risk activities for noise-induced hearing loss. Fitness instructors have experienced fluctuating hearing loss and tinnitus.^184^

Listening to music during sports activities enhances attention, reduces fatigue, and increases arousal. The more pleasurable a song is, the louder people wish to listen to it.^185^ Athletes perceive loud music as more pleasurable than less intense sounds.^186^

Loud music features 3 main elements of addictive substances, as it causes rapid and intense changes in mood and arousal level, reduces negative mood states, and makes individuals wish to listen to the music again.^183^ Prevention campaigns in gyms and for athletes focusing on the risks of loud music are rare.^187^ Listening to music during physical activity is associated with the risk of hearing loss, particularly when the activity is performed indoors.

Niskar et al.^188^ described the prevalence of hearing loss in US children aged 6 to 19 years and showed that 14.9% of 6166 participants had low-frequency or high-frequency hearing loss of at least 16 dB. Shargorodsky et al.^189^ compared the prevalence of hearing loss in adolescents aged 12 to 19 years between 2 different time periods. In 1988‒1994, 14.9% of the adolescents had hearing loss, whereas in 2005‒2006, 19.5% had hearing loss. Tung et al.^190^ found that 11.9% of 1878 first-year students at a university in Taiwan had unilateral or bilateral hearing thresholds above 25 dB. Balanay et al.^191^ reported that 39.6% of 2151 college students experienced at least one hearing symptom.

A study evaluating 5249 US adolescents aged 12 to 19 years showed that 15.9% had hearing deficits attributable to noise exposure.^7^ In the Netherlands, 14.2% of 5355 children aged 9 to 11 years had hearing impairment possibly correlated with noise-induced hearing loss.^8^ In Poland, 11.5% of 643 youths aged 13 to 18 years had hearing loss at 4 or 6 kHz, and it was significantly higher in those with heavy exposure to loud music (16.3%) than in those with mild exposure (10.7%).^192^

Exposure to moderate levels of noise, even in the short term, can cause extensive degeneration of inner hair cell synapses, but without hair cell degeneration. Audiometric testing is normal, but when evaluating a large number of noise-exposed people, on average, the thresholds are worse than those of people not exposed to noise.^193^, ^194^ Cochlear synaptopathy can cause hidden hearing loss, which presents with a normal audiogram with difficulty in speech perception in noisy environments. Cochlear synapse degeneration may also impair auditory function in many aspects, such as neural adaptation, sound localization, and temporal speech processing. Degeneration of this synapse is observed in the early stage of age-related hearing loss and may accelerate its progression.^195^

Children and adolescents, even with minimal hearing loss, can have long-term consequences with adverse effects on language development and academic performance. Noise exposure in young people can negatively affect the auditory cortex and increase susceptibility to the effects of aging on hearing.^196^

#### Recommendations

Recreational noise exposure can cause tinnitus and hearing loss in adolescents and young adults (Strong recommendation – Moderate-quality evidence).

Hearing loss in noise-exposed populations is slow but progressive, despite normal audiometric thresholds in the first few years of exposure (Weak recommendation - High-quality evidence).

## Conclusion

The Brazilian Federal Constitution of 1988, in its article 196, defines that health is a right of all and a duty of the State. To achieve this goal, the Brazilian public national health care system (Unified Health System, or SUS for short, in Portuguese) was created in accordance with the guidelines of decentralization, comprehensive care, and popular participation, while respecting the principles of universal care, integrality, and equity.^332^ Thus, every child with suspected hearing loss has the right to diagnosis and appropriate treatment if necessary. This task force considers 5 essential rights: (1) Otolaryngologist consultation; (2) Speech assessment and therapy; (3) Diagnostic tests; (4) Treatment; (5) Ophthalmologist consultation.

National campaigns should be implemented to raise awareness of the importance of caring for and treating children with hearing loss not just at birth, because many diseases manifest later, as explained in this document. Children at risk of hearing loss with poor school performance or delayed speech development should be evaluated by an otolaryngologist, qualified to assess and indicate the best path to follow.

Speech-language pathology assessment plays a critical role in determining treatment and should be performed after evaluation by an otolaryngologist. The speech therapist should be qualified to perform the tests required for each child and, together with the otolaryngologist, indicate the best treatment option for the child.

Diagnostic tests are essential to define a child’s hearing impairment. Newborn hearing screening with automated OAE measurements should not be repeated indefinitely. Children who fail the first evaluation should undergo a retest within 30 days. The follow-up for tests should be conducted according to the flowcharts presented in this document.

Access to high-quality diagnostic tests is known to be difficult. Therefore, not only the devices required for the tests but also continuing education to audiologists and otolaryngologists should be made available. Children with external auditory canal stenosis or atresia require tests such as bone conduction ABR.

Indiscriminate laboratory testing without clinical suspicion is ineffective and has low PPV. Investigation of the most common gene causing deafness, the GJB2 gene, should be offered to families with children diagnosed with profound hearing loss, in addition to imaging (CT or MRI) according to the diagnostic suspicion and reality of each health facility.

## Funding

The authors have no financial relationships relevant to this article to disclose.

## Conflicts of interest

The authors declare no conflicts of interest.
